# Mitochondrial ATP production provides long-range control of endothelial inositol trisphosphate–evoked calcium signaling

**DOI:** 10.1074/jbc.RA118.005913

**Published:** 2018-11-29

**Authors:** Calum Wilson, Matthew D. Lee, Helen R. Heathcote, Xun Zhang, Charlotte Buckley, John M. Girkin, Christopher D. Saunter, John G. McCarron

**Affiliations:** From the ‡ Strathclyde Institute of Pharmacy and Biomedical Sciences, University of Strathclyde, SIPBS Building, 161 Cathedral Street, Glasgow G4 0RE, Scotland, United Kingdom and; the § Centre for Advanced Instrumentation, Biophysical Sciences Institute, Department of Physics, Durham University, South Road, Durham DH1 3LE, United Kingdom

**Keywords:** endothelial cell, inositol 1,4,5-trisphosphate (IP3), calcium imaging, endoplasmic reticulum (ER), mitochondria, cell signaling, fluorescence imaging, inositol trisphosphate, mesenteric artery, reactive oxygen species (ROS), endothelium, ATP, Ca2+ signaling, myoendothelial junction

## Abstract

Endothelial cells are reported to be glycolytic and to minimally rely on mitochondria for ATP generation. Rather than providing energy, mitochondria in endothelial cells may act as signaling organelles that control cytosolic Ca^2+^ signaling or modify reactive oxygen species (ROS). To control Ca^2+^ signaling, these organelles are often observed close to influx and release sites and may be tethered near Ca^2+^ transporters. In this study, we used high-resolution, wide-field fluorescence imaging to investigate the regulation of Ca^2+^ signaling by mitochondria in large numbers of endothelial cells (∼50 per field) in intact arteries from rats. We observed that mitochondria were mostly spherical or short-rod structures and were distributed widely throughout the cytoplasm. The density of these organelles did not increase near contact sites with smooth muscle cells. However, local inositol trisphosphate (IP_3_)-mediated Ca^2+^ signaling predominated near these contact sites and required polarized mitochondria. Of note, mitochondrial control of Ca^2+^ signals occurred even when mitochondria were far from Ca^2+^ release sites. Indeed, the endothelial mitochondria were mobile and moved throughout the cytoplasm. Mitochondrial control of Ca^2+^ signaling was mediated by ATP production, which, when reduced by mitochondrial depolarization or ATP synthase inhibition, eliminated local IP_3_-mediated Ca^2+^ release events. ROS buffering did not significantly alter local Ca^2+^ release events. These results highlight the importance of mitochondrial ATP production in providing long-range control of endothelial signaling via IP_3_-evoked local Ca^2+^ release in intact endothelium.

## Introduction

The classical view of mitochondria is that the organelles are the “battery” of the cell, which cater for cellular energy requirements by producing ATP. However, this is not always the case. Some cells (*e.g.* cancer cells) rely on glycolysis to meet energy requirements, and others (*e.g.* erythrocytes) contain no mitochondria. Endothelial cells (ECs)^3^ form the innermost layer of the vasculature and are in direct contact with circulating blood. As such, endothelial cells are usually exposed to a nutrient- and oxygen-rich environment. Despite the abundant supply of oxygen and mitochondrial substrates, ECs reportedly do not rely on mitochondrial ATP production to meet the cells' major energy demands. Instead, energy is seemingly derived from glycolysis (1–6). Several proposals may explain why endothelial cells may rely on glycolysis in aerobic conditions (7) (like the Warburg effect in cancer cells (8)). For example, decreased oxidative phosphorylation may preserve oxygen for transfer to vascular smooth muscle and perivascular cells. Alternatively, the increased speed of ATP generation via glycolysis *versus* oxidative phosphorylation may enable ECs to meet rapid changes in energy demands. As endothelial cells are required to grow into hypoxic surroundings during angiogenesis, a reliance on anaerobic metabolism may enable ECs to form new vessels. These observations have led to the proposal that endothelial mitochondria act primarily as essential signaling organelles rather than being energy providers (9, 10).

However, despite the prevailing view of endothelial cells as a “glycolytic” cell type (11), a number of studies have suggested an important role for mitochondrial ATP generation in the endothelium. For example, several studies have suggested that glutamine and fatty acid oxidation are the main source of ATP in endothelial cells (12). Others have demonstrated that mitochondrial uncouplers inhibit angiogenesis (13). Together, the conflicting observations suggest that differential activation of the various endothelial metabolic pathways may occur under conditions of stress or glucose deprivation (4, 14, 15).

Many endothelial cell functions, such as the production of vasoactive substances (*e.g.* NO, prostacyclin, endothelium-derived hyperpolarizing factor, and endothelin), adhesion molecules (*e.g.* von Willebrand factor), and clotting factors, occur in a Ca^2+^-dependent manner. In various other cell types, Ca^2+^ signals are regulated by mitochondria. Uptake of the ion by mitochondria may promote Ca^2+^ release from IP_3_R (16–24), limit IP_3_-evoked Ca^2+^ signals (25, 26), or slow IP_3_-evoked Ca^2+^ wave progression (27–32). Mitochondria also regulate spontaneous Ca^2+^ events arising from the ryanodine receptor (33, 34). For mitochondrial Ca^2+^ uptake to control Ca^2+^ signals, it is a requirement that mitochondria be positioned close to release channels because of the low affinity of the uniporter for Ca^2+^. Indeed, mitochondria may be tethered to within 10 nm of the internal Ca^2+^ store (35, 36). At sites of close contact, channels on the internal Ca^2+^ store and mitochondrial channels (*e.g.* the uniporter and voltage-dependent anion-selective channel) may cluster (37–39). In smooth muscle, mitochondrial Ca^2+^ uptake is fast enough to regulate local Ca^2+^ signals arising from IP_3_Rs (Ca^2+^ puffs) (40), demonstrating tight functional coupling between IP_3_Rs and mitochondria. Increasing the extent of linkage between the internal Ca^2+^ store and mitochondria, by expressing a synthetic tether, increases the coupling between endoplasmic reticulum (ER) Ca^2+^ release and mitochondrial Ca^2+^ uptake in RBL-2H3 cells. Conversely, disrupting the linkage by limited proteolysis decreases mitochondrial Ca^2+^ uptake (36). These findings point to mitochondrial control of Ca^2+^ signaling arising from close coupling of the organelle and internal Ca^2+^ store and highlight the importance of the structure and position of mitochondria in regulating Ca^2+^ release events.

In native murine endothelial cells, spontaneous Ca^2+^ release events arising from the ER may occur preferentially at sites of contact between endothelial cells and smooth muscle cells (myoendothelial projections (MEPs)) (42). Ca^2+^ signals at these sites are reported to be distinctive among Ca^2+^ signals, being tightly confined (∼15 μm^2^), rapid (∼0.25 s) events arising from IP_3_Rs and referred to as pulsars (42). MEPs themselves are restricted spaces that contain an abundance of ER and proteins that govern smooth muscle cell function (*e.g.* hemoglobin α and nitric-oxide synthase (43), IP_3_Rs (42), and Ca^2+^-activated K^+^ channels (44)). Localized Ca^2+^ signaling at the MEP directly couples to these Ca^2+^-sensitive processes to control vascular function. Mitochondria critically regulate endothelial Ca^2+^ responses to shear stress activation (45, 46) and may contribute to the activation of nitric-oxide synthase (47), raising the prospect of preferential control of Ca^2+^ signaling at these sites by the organelles. However, whether or not endothelial Ca^2+^ signaling is controlled by mitochondria at the MEP is unresolved, and, indeed, little is known about mitochondrial control of endothelial Ca^2+^ signaling in intact tissues (45).

To address this issue, we examined spontaneous Ca^2+^ release events in endothelial cells in intact blood vessels obtained from rats. We found that local endothelial Ca^2+^ signals preferentially initiate at contact sites with smooth muscle cells, and the Ca^2+^ signals share the pharmacological profile of Ca^2+^ pulsars. However, the local signals have a substantially different temporal profile from pulsars. The occurrence of local Ca^2+^ signals at MEPs requires polarized mitochondria. Inhibition of mitochondrial respiration eliminates Ca^2+^ activity at MEPs. Surprisingly, neither mitochondrial positioning nor density correlates with sites that give rise to cytosolic Ca^2+^ events, as might be predicted if tethering were required for mitochondrial control of Ca^2+^ release. Ca^2+^ event initiation sites are, on average, 0.9 μm from the nearest mitochondrion and on occasion up to 5 μm away. These results indicate that close coupling is not required for mitochondrial control of Ca^2+^ release in the endothelium and that control is exerted over distance. Inhibition of the ATP synthase eliminated local Ca^2+^ signaling events, whereas buffering reactive oxygen species had little effect. Thus, mitochondria exert long-range control of IP_3_-mediated intracellular Ca^2+^ signaling dynamics in native endothelial cells via ATP production.

## Results

### Imaging the endothelium

**Figure 1.**
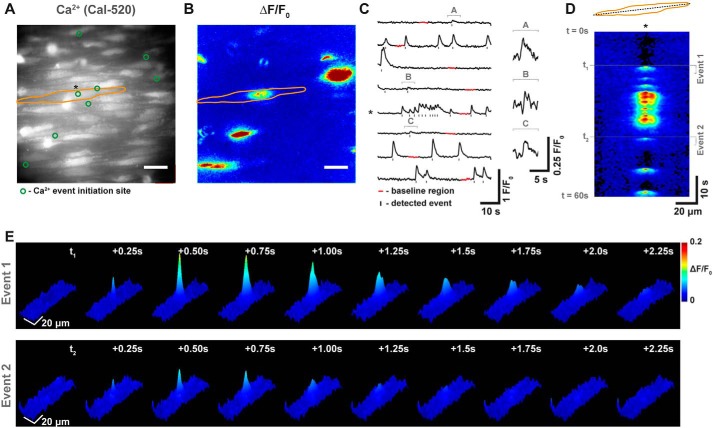
**Spontaneous Ca^2+^ signaling in native mesenteric endothelial cells.** *A* and *B*, fluorescence image (*A*) and *pseudocolored* Δ*F*/*F*_0_ maximum intensity projection (*B*) of a single field of endothelial cells of an *en face* rat mesenteric artery (∼150-μm diameter) loaded with the fluorescence Ca^2+^ indicator, Cal-520/AM, and imaged at 20 Hz. In *A* and *B*, the *orange outline* demarcates a single endothelial cell, and the *green circles* shown in *A* indicate initiation sites of Ca^2+^ activity. *Scale bars*, 20 μm. *C*, fluorescence (*F*/*F*_0_) traces from the initiation sites indicated in *A*. *, trace from the similarly marked imitation site in *A*. Some rises in [Ca^2+^]*_i_* are large Ca^2+^ events (waves) that traverse through part of individual cells, whereas other Ca^2+^ events are more localized and rapid. On rare occasions, some Ca^2+^ events appear to spread to neighboring endothelial cells (*bottom left*). The scale in *C* has been optimized to show traces that originate from large Ca^2+^ events. Shown on this scale, some traces appear to lack Ca^2+^ activity. However, on an expanded scale (*inset*), events can be clearly visualized. *D*, a two-dimensional kymograph (*line scan*) showing signal intensity (*color*) plotted against time (*y* axis) for the corresponding line drawn the length of the cell outlined in *A*. Again, an *asterisk* indicates the position of the Ca^2+^ initiation site within this cell. Events of varying amplitudes and spatial spreads arise from the single initiation site. *E*, three-dimensional surface plots show that two of these events (marked in *D*) are of markedly different amplitudes/spreads. Data are also shown in Movie S1.

Ca^2+^ signaling was assessed in endothelial cells of intact second-order mesenteric arteries (∼150-μm diameter). Time-series image recordings (20 Hz) of the endothelium were obtained using a high-NA (1.3) ×100 microscope objective and a large-format EMCCD camera (1024 × 1024 pixels; 13-μm pixel size). In opened arteries (*en face* preparations), this experimental set-up provided a field of view of ∼17,720 μm^2^ with a pixel size of 130 × 130 nm projected onto the endothelium. On average, 52 ± 2 whole or partial endothelial cells were visualized in each field of view (Fig. 1*A*; 27 fields from *n* = 9 animals). Thus, we calculated the density of endothelial cells to be on the order of 2000 cells/mm^2^, in agreement with our previous estimates (48).

### Spontaneous endothelial calcium signaling

In the absence of stimulation with pharmacological agents or mechanical forces, close visual inspection of raw (Fig. 1*A* and Movie S1) and baseline-corrected (*F*/*F*_0_; Fig. 1*B* and Movie S2) fluorescence recordings revealed extensive Ca^2+^ activity in mesenteric artery endothelial cells. The signals formed a continuum of events that ranged from small, highly localized focal increases in Ca^2+^ (akin to Ca^2+^ puffs) to traveling spatial gradients (waves) that progressed completely or partly (partial Ca^2+^ waves) through cells. Partial Ca^2+^ waves were the predominant form of Ca^2+^ activity. On average, 12.5 ± 1.5% of ECs exhibited spontaneous Ca^2+^ activity (27 fields from *n* = 9 animals). Often, a single location would give rise to repetitive Ca^2+^ events, although the properties (*e.g.* magnitude, spatial spread) of events that arose from single sites varied (Fig. 1, *C–E*).

### Characteristics of basal calcium events

**Figure 2.**
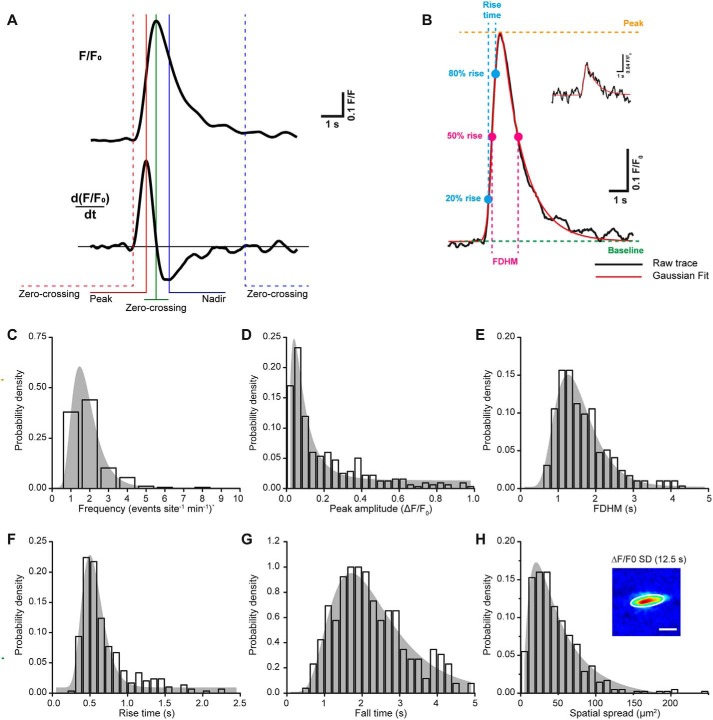
**Basal Ca^2+^ events form a continuum of signaling events.** *A*, a Ca^2+^ trace (*top*) and corresponding derivative (*bottom*) illustrating the automated peak detection method used to identify Ca^2+^ events. *B*, a large and small (*inset*) Ca^2+^ event (*black line*) together with a fit to an exponentially modified Gaussian model (*red line*). Principal measurements of the Ca^2+^ trace (baseline (*green line*), peak (*orange line*), FDHM (*magenta lines* and *cursors*), and rise time (*t*_20_–*t*_80_) (*cyan lines* and *cursors*)) were used to provide appropriate conditions to the model-fitting algorithm. *C–H*, probability density histograms of frequency of events at each site (*C*), peak amplitude (*D*), FDHM (*E*), rise time (*F*), fall time (*G*), and spatial spread (*H*). *Inset*, the extent to which Ca^2+^ event spread was calculated by fitting a two-dimensional Gaussian (*white outline*) to an S.D. intensity projection of Δ*F*/*F*_0_ image sequences. *Scale bar*, 20 μm.

To analyze spontaneous local endothelial Ca^2+^ activity, we manually identified (from *F*/*F*_0_ representations of Ca^2+^ recordings) the initiation sites from which Ca^2+^ activity originated and applied an automated Ca^2+^ signal analysis algorithm. The algorithm was adapted from our previous work (45) (Fig. 2*A*; also see “Experimental procedures”) For each initiation site, we extracted baseline-corrected Ca^2+^ signals (*F*/*F*_0_; Fig. 1*C*) and then automatically identified Ca^2+^ events (Fig. 2*A*). The algorithm identifies Ca^2+^ events using peaks in the time derivative of the *F*/*F*_0_ signal. Ca^2+^ events were taken as changes in the time derivative of the *F*/*F*_0_ signal that exceeded a threshold value of 10 times the S.D. value of the baseline signal fluctuation. This threshold (10-fold baseline noise) was chosen empirically, as being large enough to exclude noise fluctuations but small enough to faithfully detect low-amplitude Ca^2+^ signals. A suitable baseline period was automatically identified for each trace (*red portions* of traces in Fig. 1*C*). Following event extraction, we then used a least-squares optimization algorithm to fit an exponentially modified Gaussian function (Fig. 2*B*, *red line*) to each Ca^2+^ peak to measure event parameters. Histograms (Fig. 2, *C–H*; *n* = 320 events) show that each measured parameter (event frequency, amplitude, full duration at half-maximum (FDHM), rise time, fall time, and spatial spread) may be described by a continuous log normal distribution. The mean values of each were as follows: frequency (1.71 events site^−1^ min^−1^, 95% CI 1.59–1.84 events site^−1^ min^−1^); amplitude (0.13 *F*/*F*_0_, 95% CI 0.11–0.14 *F*/*F*_0_); FDHM (1.11 s, 95% CI 1.03–1.19 s); rise time (0.55 s, 95% CI 0.51–0.58 s); fall time (1.86 s, 95% CI 1.71–2.01 s), and spatial spread (38 μm^2^, 95% CI 35–42 μm^2^).

### Pharmacological profile of basal calcium events

**Figure 3.**
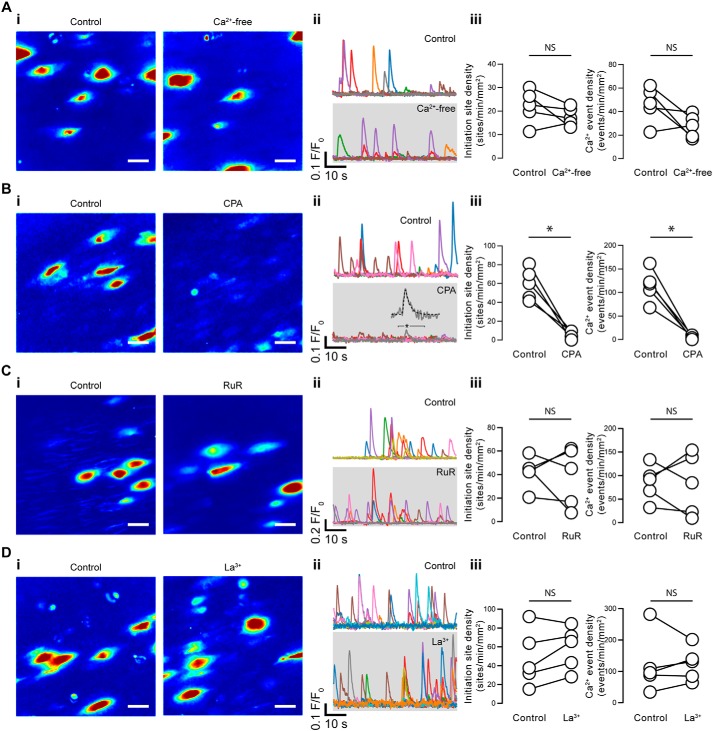
**Basal endothelial Ca^2+^ events arise via Ca^2+^ release from internal stores.** *A–D*, effects of Ca^2+^-free PSS (with 1 mm EGTA; *A*), CPA (*B*), RuR (*C*), and La^3+^ (*D*) on basal endothelial Ca^2+^ events. *i*, composite Ca^2+^ images illustrating Ca^2+^ activity (in the same field of endothelial cells) during a 1-min period before (*left*) and after (*right*) pharmacological intervention; each image pair is shown on the same intensity scale; *ii*, Ca^2+^ traces from the events shown in the corresponding panel *i*; *iii*, paired summary data showing the density of Ca^2+^ event initiation sites and Ca^2+^ events. Each data point indicates the mean of three technical replicates (three fields of endothelial cells) from a single experimental unit (one animal). *, *p* < 0.05; *NS*, no statistically significant difference detected (*i.e. p* > 0.05) using paired *t* test. *Scale bars*, 20 μm.

Spontaneous Ca^2+^ events may arise from to Ca^2+^ entry from outside the cell or as a result of release from intracellular stores. To establish the source of Ca^2+^, we performed experiments using a Ca^2+^-free bathing solution (with 1 mm EGTA; Fig. 3*A*). Removal of external Ca^2+^ had no significant effect on either the density of Ca^2+^ event initiation sites (22 ± 3 sites min^−1^ mm^−2^ for control; 18 ± 2 sites min^−1^ mm^−2^ for Ca^2+^-free; *p* = 0.20) or the density of Ca^2+^ events (46 ± 7 events min^−1^ mm^−2^ for control; 27 ± 4 events min^−1^ mm^−2^ for Ca^2+^-free; *p* = 0.09), suggesting that the events arose via Ca^2+^ release from the internal store rather than Ca^2+^ influx (15 fields from *n* = 5 animals). In support, the SERCA inhibitor, cyclopiazonic acid (CPA; 5 μm), abolished basal endothelial Ca^2+^ events (Fig. 3*B*). CPA reduced the initiation site density from 59 ± 7 to 5 ± 10 sites min^−1^ mm^−2^, whereas the event density was reduced from 115 ± 15 to 5 ± 2 events min^−1^ mm^−2^ (*p* < 0.05 for each, 15 fields from *n* = 5 animals). Ca^2+^ events that persisted after CPA incubation could still be described by an exponentially modified Gaussian function (Fig. 3*C* (*ii*), *inset*). The broad-spectrum TRPV channel antagonist, ruthenium red (RuR) (Fig. 3*C*), was without effect on either the density of Ca^2+^ event initiation sites (42 ± 25 sites min^−1^ mm^−2^ for control; 38 ± 7 sites min^−1^ mm^−2^ for RuR; *p* = 0.72) or the density of Ca^2+^ events (85 ± 17 events min^−1^ mm^−2^ for control; 82 ± 29 events min^−1^ mm^−2^ for RuR; *p* = 0.91). We have previously shown that this concentration of RuR (5 μm) is sufficient to inhibit endothelial Ca^2+^ activity induced by the specific TRPV4 agonist, GSK1016790A (45). Similarly, the nonspecific Ca^2+^ release–activated channel inhibitor, lanthanum (La^3+^) (Fig. 3*D*), also failed to inhibit basal endothelial Ca^2+^ activity (22 ± 7 sites min^−1^ mm^−2^ for control, 23 ± 4 sites min^−1^ mm^−2^ for La^3+^, *p* = 0.14; 48 ± 14 events min^−1^ mm^−2^ for control, 59 ± 10 events min^−1^ mm^−2^ for La^3+^, *p* = 0.79).

**Figure 4.**
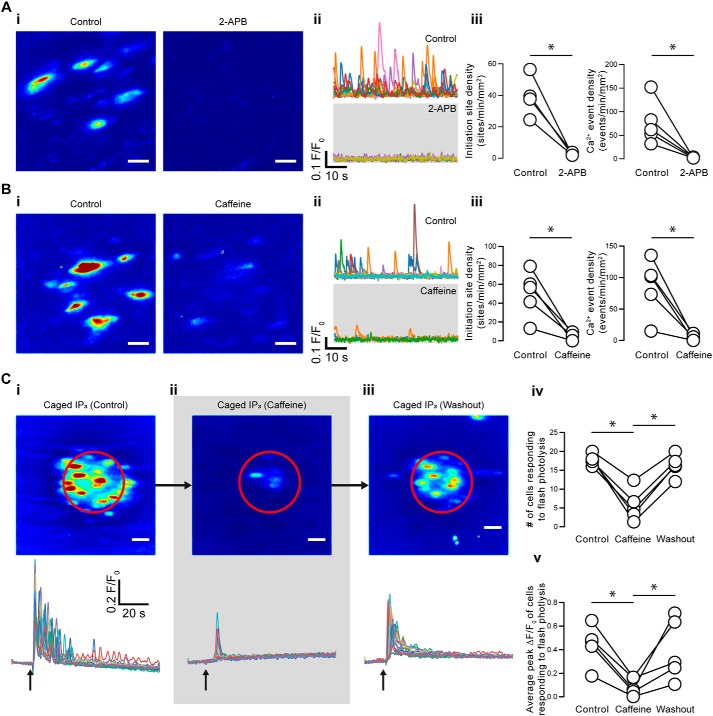
**Basal endothelial Ca^2+^ events arise via the IP_3_ receptor.** *A* and *B*, effects of 2-APB (100 μm) (*A*) and caffeine (10 mm) (*B*) on basal endothelial Ca^2+^ events. *i*, composite Ca^2+^ images illustrating Ca^2+^ activity (in the same field of endothelial cells) during a 1-min period before (*left*) and after (*right*) pharmacological intervention; *ii*, Ca^2+^ traces from the events shown in the corresponding panel *i*; *iii*, paired summary data showing the density of Ca^2+^ event initiation sites and Ca^2+^ events. Each data point indicates the mean of three technical replicates (three fields of endothelial cells) from a single experimental unit (one animal). *C*, effect of caffeine on Ca^2+^ signals evoked by local photolysis of caged IP_3_. *i–iii*, composite Ca^2+^ images (*top*) and superimposed single-cell Ca^2+^ traces (*bottom*) illustrating Ca^2+^ activity in response to UV uncaging (flash region indicated by *red outline*) in the absence of caffeine (*i*), in the presence of caffeine (*ii*), and after washout of caffeine (*iii*). *iv* and *v*, paired summary data showing the number of cells that responded to photorelease of caged IP_3_ with a Ca^2+^ rise (*iv*) and the average magnitude of the peak Ca^2+^ signal in responding cells. Each data point indicates the mean from a single field of endothelial cells (one animal). *, *p* < 0.05; *NS*, no statistically significant difference detected (*i.e. p* > 0.05) using paired *t* test or repeated measures analysis of variance with Dunnett's multiple-comparison test, as appropriate. *Scale bars*, 20 μm; within each experimental series, all images are shown on the same intensity scale.

Ca^2+^ release from the endoplasmic reticulum may occur primarily via IP_3_Rs in the vascular endothelium (49). To investigate whether the continuum of Ca^2+^ activity arose from IP_3_Rs, we performed experiments using the IP_3_R inhibitor, 2-aminoethoxydiphenyl borate (2-APB; 100 μm). 2-APB reduced the Ca^2+^ event initiation site density from 36 ± 6 to 2 ± 0 sites min^−1^ mm^−2^ and the Ca^2+^ event density from 76 ± 21 to 3 ± 0 events min^−1^ mm^−2^ (*p* < 0.05 for each, 15 fields from *n* = 5 animals). Although 2-APB blocks IP_3_Rs in native endothelial cells, it may also inhibit Ca^2+^ entry pathways (45). Therefore, we used an additional IP_3_R antagonist, caffeine (10 mm; Fig. 4, *B* and *C*). Caffeine reduced the Ca^2+^ event initiation site density from 50 ± 11 to 4 ± 2 sites min^−1^ mm^−2,^ and the Ca^2+^ event density from 86 ± 20 to 5 ± 2 events min^−1^ mm^−2^ (*p* < 0.05 for each, 15 fields from *n* = 5 animals; Fig. 4*B*). As a control, we also show that caffeine inhibited Ca^2+^ release induced by photolysis of caged IP_3_ (Fig. 4*C*).

**Figure 5.**
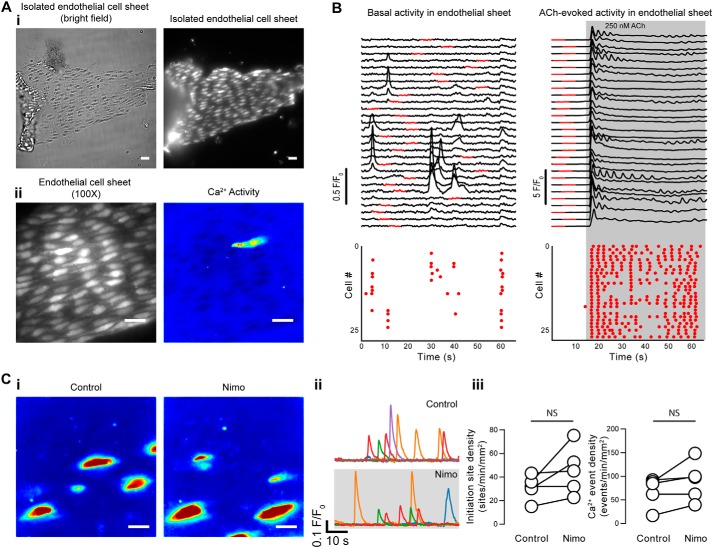
**Basal endothelial Ca^2+^ events occur independently of smooth muscle cell activity.** *A*, images of a freshly isolated sheet of endothelial cells from a second-order mesenteric artery loaded with the fluorescence Ca^2+^ indicator, Cal-520. Images in *A* (*i*) were taken at ×40 magnification and show a sheet visualized by both bright-field (*left*) and fluorescence (*right*) imaging. Images in *A* (*ii*) show raw fluorescence (*left*) and composite Ca^2+^ activity (*right*) of the same isolated endothelial cells imaged at ×100 magnification. *B*, example Ca^2+^ traces showing basal (*left*) and ACh-evoked (250 nm; *right*) Ca^2+^ signals obtained from each cell in a sheet of 28 endothelial cells. *Red dots* in the *bottom panels* indicate the time at which peaks were detected in the Ca^2+^ signals. *C*, effects of nimodipine (*nimo*; 10 μm) on basal endothelial Ca^2+^ events in endothelial cells of an intact artery. *i*, composite Ca^2+^ images illustrating Ca^2+^ activity (in the same field of endothelial cells) during a 1-min period before (*left*) and after (*right*) pharmacological intervention; *ii*, Ca^2+^ traces from the events shown in the corresponding panel *i*; *iii*, paired summary data showing the density of Ca^2+^ event initiation sites and Ca^2+^ events. Each data point indicates the mean of three technical replicates (three fields of endothelial cells) from a single experimental unit (one animal). *NS*, no statistically significant difference detected (*i.e. p* > 0.05) using paired *t* test. *Scale bars*, 20 μm.

Spontaneous Ca^2+^ events did not arise from mechanisms involving voltage-activated Ca^2+^ channels located on smooth muscle cells. In support, spontaneous Ca^2+^ events were present in isolated endothelial patches (Fig. 5*A*). Furthermore, the Ca^2+^ channel blocker, nimodipine (10 μm), did not reduce the density of initiation sites (31 ± 5 sites min^−1^ mm^−2^ for control; 46 ± 9 sites min^−1^ mm^−2^ for nimodipine; *p* = 0.13) or indeed of events themselves (69 ± 14 events min^−1^ mm^−2^ for control; 90 ± 19 events min^−1^ mm^−2^ for nimodipine; *p* = 0.12) in intact arteries (Fig. 5*B*; 15 fields from *n* = 5 animals). This concentration of nimodipine (10 μm) is sufficient to fully block voltage-dependent Ca^2+^ currents in smooth muscle cells (50–52) and to prevent depolarizing-induced (70 mm K^+^) contraction of isolated arteries (45).

Collectively, these results suggest that IP_3_-mediated Ca^2+^ events are the predominant basal Ca^2+^ signaling modality in small mesenteric arteries of the rat.

### Location of basal calcium events

In murine mesenteric arteries, transient nonpropagating IP_3_-mediated Ca^2+^ release events (Ca^2+^ pulsars) occur extensively in unstimulated endothelium (42). Pulsars also occur preferentially at MEPs. The pharmacological profiles of the Ca^2+^ events described in the present study are similar to Ca^2+^ pulsars. However, their amplitude, kinetic properties, and spatial spread differ substantially. Pulsars are brief (lasting ∼0.25 s) and confined (spread of ∼15 μm^2^) events when compared with the Ca^2+^ signals observed in the present study (Fig. 2). The differences may arise because the signaling modalities are fundamentally different or from differences in the Ca^2+^ buffer capacity of the endothelium across species or as a result of differences in experimental conditions (*e.g.* different Ca^2+^ indicators or light intensity) (53). Notwithstanding, to determine whether the continuum of Ca^2+^ events described here arises preferentially in the vicinity of MEPs, we imaged the position of Ca^2+^ event initiation sites and holes in the internal elastic lamina (IEL) where MEPs occur.

**Figure 6.**
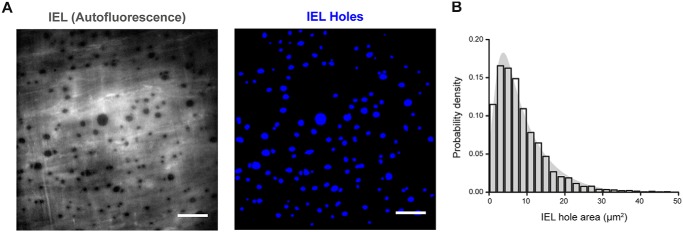
**Fenestration of the internal elastic lamina is extensive.** *A*, representative image of the IEL of an *en face* rat mesenteric artery (*left*) and processed image highlighting fenestrations (holes) (*right*). In the *left panel*, elastin has been visualized via autofluorescence, and holes are indicated by a lack of fluorescence signal (*black*). In the *right panel*, raw autofluorescence images have been processed and inverted (see “Experimental procedures”) to highlight the IEL holes (*blue*). IEL holes represent possible sites of coupling between endothelial and smooth muscle cells (myoendothelial gap junctions). *Scale bars*, 20 μm. *B*, histogram illustrating the approximately log normal distribution of the IEL hole area. The histogram shows pooled data (3971 IEL holes) from 27 fields of endothelial cells (*n* = 9). On average, 7.0 ± 0.6% of the IEL was occupied by fenestrations (*n* = 9).

The IEL of intact small mesenteric arteries was visualized using autofluorescent emission from the elastin layer upon UV illumination. In autofluorescence images, holes in the IEL appear as *dark regions* in the fluorescence field (Fig. 6*A*, *left*). To highlight the location of these IEL holes, we smoothed, inverted, and colorized autofluorescence images so that IEL holes were shown as *blue* on a *black background* (Fig. 6*A*, *right*). IEL holes were distributed extensively across the IEL, with 819 ± 58 holes/mm^2^ corresponding to a fenestrated area of 7.0 ± 0.6% of the elastic lamina. This measurement is similar to the estimate in murine mesenteric arteries (42) but substantially larger (nearly 2 orders of magnitude) than that in another study (54). The average size of IEL holes was 8.7 ± 0.8 μm^2^ (*n* = 27 fields; *n* = 9). A pooled analysis of all IEL holes observed in 27 fields of endothelial cells (*n* = 9) demonstrated the lognormal distribution of IEL hole area (Fig. 6*B*; *n* = 3971 IEL holes).

**Figure 7.**
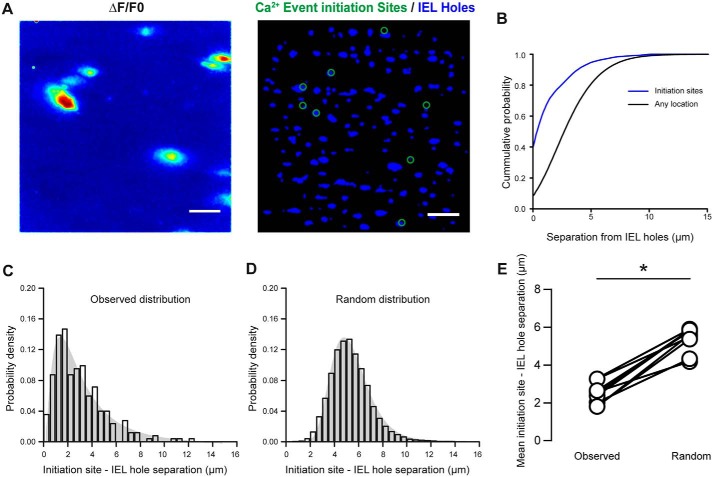
**Basal endothelial Ca^2+^ events initiate preferentially at sites of IEL holes.** *A*, *pseudocolored* Ca^2+^ image (Δ*F*/*F*_0_ maximum intensity projection; *left*) and a composite image (*right*) showing IEL holes (*blue*) from the same field of endothelium with Ca^2+^ event initiation sites (*green circles*) overlaid. *Scale bars*, 20 μm. *B*, cumulative probability distributions illustrating the tight coupling of Ca^2+^ event initiation sites to IEL holes. *C*, histogram illustrating the distribution (log normal) of the centroid–centroid distance between Ca^2+^ event initiation sites and IEL holes. *D*, histogram displaying a random distribution (1000 permutations) of the average distance between Ca^2+^ event initiation sites and the nearest IEL hole calculated for the data shown in *A* and *B. E*, the centroid–centroid distance (paired data points) between Ca^2+^ event initiation sites and the nearest IEL observed (2.4 ± 0.1 μm) was significantly lower than expected from a randomized distribution of Ca^2+^ event initiation sites (5.1 ± 0.2 μm; *n* = 27 fields, *n* = 9; Student's paired *t* test; *, *p* < 0.05).

Fig. 7*A* shows a typical image obtained when Ca^2+^ event initiation sites from a field of endothelial cells (identified in *F*/*F*_0_ recordings) are overlaid on an image of the underlying IEL holes. In all 27 fields in which endothelial Ca^2+^ and the underlying IEL were investigated, Ca^2+^ events occurred close to IEL holes (Fig. 7*A*). Measuring the distance from each event initiation site to the nearest IEL hole revealed that, on average, ∼50% (*n* = 225 distinct event sites) of Ca^2+^ events initiated directly at an IEL hole location (centroids separated by less than 2.5 μm; Fig. 7*C*), and ∼85% initiated with 5 μm of an IEL hole.

However, fenestration of the IEL is extensive, and the apparent co-localization may be expected from a random distribution of IEL holes and local Ca^2+^ signals. We thus next investigated whether or not local Ca^2+^ events occurred more often at holes in the IEL than would be predicted from a random overlap of the two. To this end, two separate tests were performed to determine whether Ca^2+^ events were statistically more likely to occur close to an IEL hole than elsewhere in the cell.

First, we pooled all data to generate (and then compare) cumulative probability distributions for the distances between initiation sites and IEL holes and the distances between all locations and IEL holes. In this analysis, we found that the separation between IEL holes and initiation sites was significantly less than the separation between IEL holes and all other sites (Fig. 7*B*; *p* < 0.05, two-sample Kolgorov–Smirnov test). This result suggests that Ca^2+^ events are more likely to occur close to an IEL than elsewhere in the cell.

Next, we performed Monte Carlo simulations (permutation tests) to generate random distributions of Ca^2+^ event initiation sites and analyzed these data. For each data set, the location of observed Ca^2+^ events was randomly redistributed 1000 times (Fig. 7, *C–E*). This resulted in 1000 sets of random Ca^2+^ event locations, and for each 1000 permutations, the centroid–centroid distances were once again measured. The average minimum distance between an IEL hole and a randomly redistributed event initiation site (5.3 μm, 95% CI 4.8–5.9 μm) was significantly higher than that measured from the real data (2.4 μm, 95% CI 2.0–2.9 μm; Fig. 5*E*; *p* < 0.05; *n* = 27, *n* = 9). Thus, spontaneous Ca^2+^ events occurred more often near IEL holes than would be expected if the Ca^2+^ events initiated randomly throughout the cytoplasm.

Taken together, the results presented thus far suggest that the Ca^2+^ events described are IP_3_-mediated Ca^2+^ pulsars, albeit with a different kinetic profile from those occurring in murine mesenteric artery. These events occur at initiation sites that are closer to MEPs (IEL holes) than expected from a random distribution. However, the large mean distance between MEPs and initiation sites (2.4 μm) suggests that direct coupling of initiation sites to MEPs is unlikely. It may be that in rat mesenteric arteries, Ca^2+^ events manifest as local propagating waves to couple activity at the initiation site to effector proteins that are located within MEPs.

### Mitochondrial control of basal endothelial Ca^2+^ signaling

**Figure 8.**
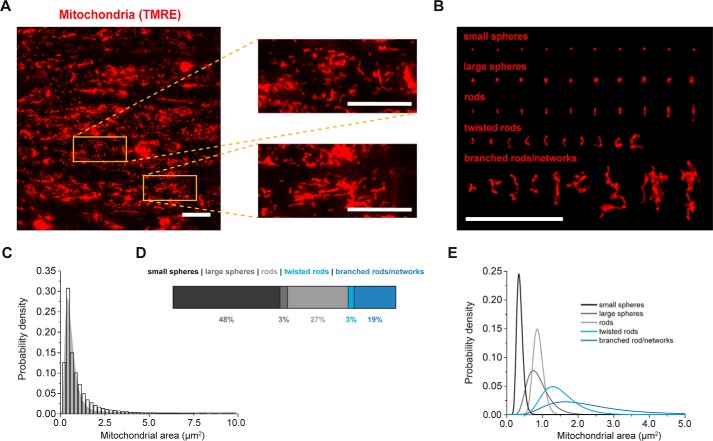
**Mitochondria in endothelial cells of intact arteries are morphologically heterogeneous.** *A*, representative fluorescence image of mitochondria, in mesenteric artery endothelium, visualized using the fluorophore TMRE (240 nm) at ×100 magnification (130-nm pixel size at object plane). Endothelial cell mitochondria are dense and occupy 8.7 ± 0.2% of the two-dimensional field-of-view (*n* = 24 fields, *n* = 8). *Insets*, expanded regions (*orange boxes*), where individual mitochondria can be resolved. *B*, image showing representative subtypes of mitochondria. *Scale bars* in *A* and *B*, 20 μm. *C*, histogram illustrating the log normal distribution of mitochondrial size (μm^2^; total of 35,541 mitochondria). *D*, horizontal bar graph illustrating the percentages of subtypes that contribute to the overall mitochondrial complement. *E*, mitochondrial size (μm^2^) distributions for each subtype. Mitochondria are mainly rods and spheres.

Mitochondria modulate Ca^2+^ signaling in a variety of excitable and nonexcitable cell types (55–57). Both the position and morphology of the organelle are critical to mitochondrial control of Ca^2+^ signals, and the organelles are reported to be close to MEPs (58). Therefore, we next investigated whether mitochondria modulate basal endothelial Ca^2+^ activity. As a first step, mitochondrial morphology was examined using the indicator tetramethylrhodamine ethyl ester (TMRE) (240 nm) to visualize the organelles (23, 59, 60). Mitochondria, in small mesenteric artery endothelial cells, were morphologically heterogeneous and were observed as small spheres, globules, and rods as well as looped (twisted) and branched rods (Fig. 8). Mitochondria did not appear to form extensive reticular networks (Fig. 8; see also Ref. 45). Mitochondrial distribution was denser in the perinuclear region, and, on average, mitochondria occupied 8.7 ± 0.2% of cell area (*n* = 24 fields, *n* = 8). This value is similar to that observed in native smooth muscle cells (7% of the cell volume (60)).

In many cell types, mitochondria are highly dynamic organelles. In others, mitochondria remain stationary when observed for extended periods (59–62). Mitochondria may promote Ca^2+^ signaling via a Ca^2+^-dependent feedback process operating between the organelle and the IP_3_ receptor (16, 25, 40, 41, 52, 63). Such feedback requires that mitochondria be positioned near Ca^2+^ release channels because the affinity of the uniporter for Ca^2+^ is low and cytosolic gradients of free [Ca^2+^] around an open channel or cluster of channels are extremely steep, falling from tens of μm or more near a channel mouth to tens of nm only a few hundred nm away (64). Thus, we next investigated whether mitochondria are located close to sites of Ca^2+^ event initiation.

**Figure 9.**
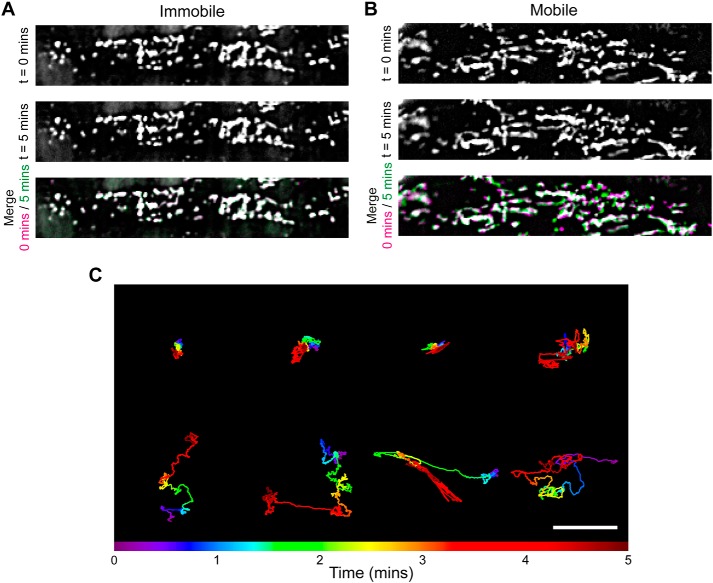
**Mitochondrial motility in native endothelial cells.** *A* and *B*, representative fluorescence images of mitochondria, in mesenteric artery endothelium, visualized using the fluorophore TMRE (240 nm). *Panels* show fluorescence images taken 5 min apart (*top* and *middle*) and a composite overlay of the two (*bottom*), where any *green* or *magenta* indicates differences in mitochondrial position between the two time point images, and *white* indicates no difference. The images in *A* show the mitochondria in a full endothelial cell in which no mitochondrial movement was observed. The images in *B* show a different preparation in which extensive movement occurred. *C*, motion tracks of eight example endothelial mitochondria, illustrating a range of motion over a 5-min period. The plot shows the relative *x-y* position of each mitochondrion at the time point indicated by the *color bar. Scale bar*, 1 μm.

As a first step in examining the relationship between the location of mitochondria and Ca^2+^ release sites, we measured the extent of mitochondrial movement in native endothelium. Over substantial imaging durations (30 min, *n* = 5), we observed extensive movement of the majority of endothelial mitochondria (Movie S3). In recordings of shorter duration (5 min), mitochondrial motion was less pronounced but was observed nonetheless in two of six recordings (Fig. 9, *A–C*). The period for Ca^2+^ imaging was 1 min. The findings suggest that mitochondria in native endothelial cells are largely mobile structures and so are unlikely to be tethered to Ca^2+^ release sites.

**Figure 10.**
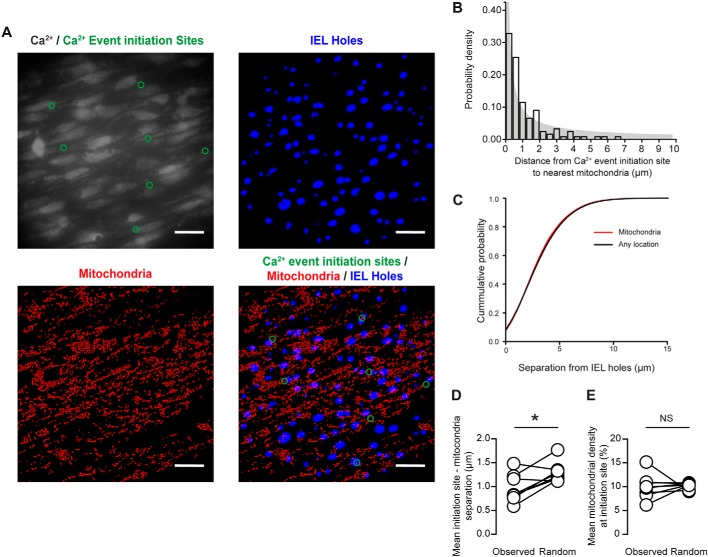
**Mitochondria are not tightly coupled to local Ca^2+^ events that initiate at MEPs.** *A*, representative images of endothelial cells (*top left*), IEL holes (*top right*), mitochondria (*bottom left*), and all three superimposed (*bottom right*) in the intact, native endothelium. *Scale bars*, 20 μm. *B*, histogram illustrating the mitochondria-Ca^2+^ event initiation site separation. *C*, cumulative probability distributions illustrating the loose coupling of Ca^2+^ mitochondria to IEL holes. *D*, mean separation of the center of Ca^2+^ event initiation sites and the nearest mitochondria (paired data points overlaid) was significantly lower than that measured from a randomized distribution of Ca^2+^ event initiation sites (*n* = 24 fields from *n* = 8 animals). *E*, mean mitochondrial density (percentage of area occupied by mitochondria) within circular areas (5-μm diameter) surrounding Ca^2+^ event initiation sites was not significantly different from a randomized distribution of Ca^2+^ event initiation sites (*n* = 24 fields, *n* = 8). *, *p* < 0.05; *NS*, no statistically significant difference detected (*i.e. p* > 0.05) using paired *t* test.

Nevertheless, we examined the relationship between mitochondrial position and Ca^2+^ release event initiation sites. Because of the possibility of mitochondrial movement, we dual loaded the endothelium with the Ca^2+^ indicator, Cal-520/AM (5 μm), and TMRE (240 nm) to enable us to record both Ca^2+^ activity and mitochondrial position from the same field of endothelial cells (Fig. 10).

During short imaging sessions (1-min duration), we observed that mitochondria appeared to be positioned close to many Ca^2+^ event initiation sites (Fig. 10, *A* and *B*). However, the separation between IEL holes (where Ca^2+^ events occur) and mitochondria was not significantly different from the separation measured between IEL holes and all other sites (Fig. 10*C*; *p* > 0.05, two-sample Kolgorov–Smirnov test). This result suggests that mitochondria are not coupled to Ca^2+^ release sites. Notably, a substantial percentage of Ca^2+^ release sites (9%) had no mitochondria located within a 2.5-μm radius, and ∼2% of Ca^2+^ release sites had no mitochondria within a 5-μm radius (from the center of initiation site to the nearest mitochondrial pixel; Fig. 10*B*, *n* = 161 sites). The averaged separation between local Ca^2+^ signal initiation sites and nearest mitochondria was slightly lower (0.9 μm, 95% CI 0.7–1.2 μm) than expected from a random distribution of event initiation sites (1.3 μm, 95% CI 1.1–1.5 μm; Fig. 10*D*; *p* < 0.05; *n* = 8). However, there was no correlation between sites of Ca^2+^ event initiation and mitochondrial density (9.7%, 95% CI 7.8–12.0% for observed data; 10.1%, 95% CI 9.6–10.7% for random data; Fig. 10*E*; *p* = 0.65; *n* = 8). The large mean separation (0.9 μm) between mitochondria and Ca^2+^ event initiation sites strengthens the view that direct modulation of Ca^2+^ initiation site activity by mitochondria is unlikely.

**Figure 11.**
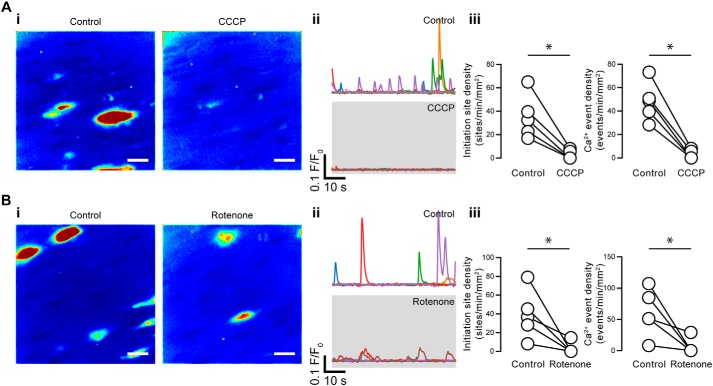
**Basal IP_3_-mediated endothelial Ca^2+^ signaling requires polarized mitochondria.** *A* and *B*, effects of CCCP (*A*) and rotenone (*B*) on basal endothelial Ca^2+^ events. *i*, composite Ca^2+^ images illustrating Ca^2+^ activity (in the same field of endothelial cells) before (*left*) and after (*right*) pharmacological intervention. Each image pair is shown on the same intensity scale; *ii*, Ca^2+^ traces from the events shown in the corresponding panel *i*; *iii*, paired summary data showing the density of Ca^2+^ event initiation sites (*left*) and Ca^2+^ events (*right*). Each data point indicates the mean of three technical replicates (three fields of endothelial cells) from a single experimental unit (one animal). *, *p* < 0.05; *NS*, no statistically significant difference detected (*i.e. p* > 0.05) using paired *t* test.

To determine the role of mitochondria in modulating basal endothelial cell Ca^2+^ signaling, we investigated the effects of the mitochondrial uncoupler, CCCP (5 μm), or the complex 1 inhibitor, rotenone (2 μm). CCCP reduced the initiation site density from 35 ± 8 to 3 ± 2 sites min^−1^ mm^−2^, whereas the event density was reduced from 48 ± 7 to 3 ± 2 events min^−1^ mm^−2^ (Fig. 11*A*; *n* = 5). Rotenone reduced the Ca^2+^ event initiation site density from 39 ± 7 to 3 ± 3 sites min^−1^ mm^−2^ and the Ca^2+^ event density from 60 ± 17 to 17 ± 6 events min^−1^ mm^−2^ (Fig. 11*B*; *n* = 5). When the proton gradient across the mitochondrial membrane is impaired by mitochondrial inhibitors, such as CCCP or rotenone, the ATP synthase may reverse and consume ATP. Therefore, in the next series of experiments, the effects of the mitochondrial ATP synthase inhibitor, oligomycin (6 μm), on the CCCP-induced decreases in basal endothelial Ca^2+^ activity were examined. Oligomycin by itself was without effect on the mitochondrial membrane potential, as assessed by TMRE fluorescence (5.1 ± 6.8% *increase* in 5 min for control, 10.8 ± 8.8% *increase* in 5 min for oligomycin, *p* = 0.70, *n* = 5, Fig. S1). In contrast, when CCCP was subsequently added, there was a rapid loss of punctate mitochondrial staining and a significant decrease in TMRE fluorescence (65.5% ± 4.1 *decrease* in 5 min for CCCP; *p* < 0.05, *n* = 5; Fig. S1). Rotenone also significantly depolarized mitochondria, as revealed by a reduction in TMRE fluorescence (6.1 ± 8.3% *decrease* in 5 min for control, 28.4 ± 5.9% *decrease* in 5 min for rotenone; *p* < 0.05, *n* = 3; Fig. S2), consistent with its role as a mitochondrial complex 1 inhibitor (65–67). Rotenone-induced depolarization was of a slower time course, when compared with CCCP, because of the different modes of action of each drug. CCCP is a protonophore that rapidly collapses the mitochondrial membrane potential. Rotenone is a complex I inhibitor that results in a slower “run-down” of the membrane potential.

**Figure 12.**
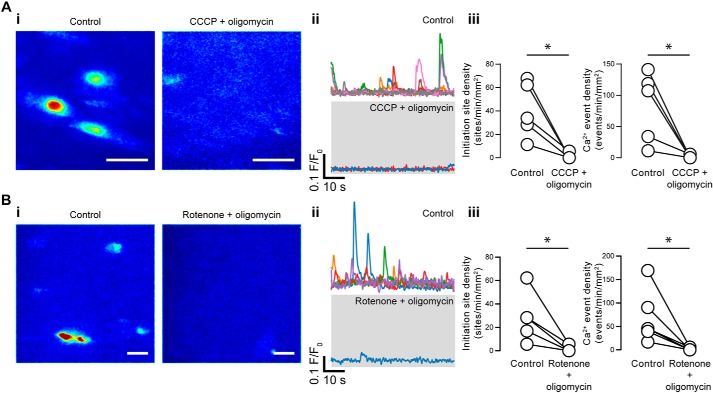
**Oligomycin does not prevent the reduction in Ca^2+^ activity caused by CCCP or rotenone.** *A* and *B*, effects of oligomycin used in combination with either CCCP (*A*) or rotenone (*B*) on basal endothelial Ca^2+^ events. *i*, composite Ca^2+^ images illustrating Ca^2+^ activity (in the same field of endothelial cells) before (*left*) and after (*right*) pharmacological intervention. Each image pair is shown on the same intensity scale; *ii*, Ca^2+^ traces from the events shown in the corresponding panel *i*; *iii*, paired summary data showing the density of Ca^2+^ event initiation sites and Ca^2+^ events. Each data point indicates the mean of three technical replicates (three fields of endothelial cells) from a single experimental unit (one animal). *, *p* < 0.05 using paired *t* test. *Scale bars*, 20 μm.

When applied in combination with CCCP, oligomycin did not prevent the reduction in Ca^2+^ activity seen when CCCP was applied by itself (Fig. 12*A*). Nor did oligomycin prevent the reduction in Ca^2+^ activity seen when rotenone was applied by itself (Fig. 12*B*).

To test whether the reduction in Ca^2+^ activity caused by these mitochondrial toxins arose from depletion of intracellular Ca^2+^ stores, we examined the Ca^2+^ response to the ionophore, ionomycin, in a Ca^2+^-free physiological saline solution (PSS) (Fig. S3). The ionomycin releasable store content was unaffected by rotenone (with oligomycin present) or oligomycin applied alone. However, the ionomycin-evoked Ca^2+^ increase was significantly reduced by CCCP (with oligomycin present). It is possible that CCCP may inhibit Ca^2+^ release from the store, as has been shown previously for other nonexcitable cells (68). However, ionomycin facilitates the transport of Ca^2+^ across the internal store by exchanging H^+^ (69, 70) from the internal store. CCCP is likely to collapse the proton gradient across the internal store. In these circumstances, CCCP may be expected to reduce ionomycin-evoked Ca^2+^ release. That neither rotenone nor oligomycin reduced the response to ionomycin suggests that the store content is unaltered by each of these interventions.

### Long-distance regulation of constitutive IP_3_-mediated Ca^2+^ signaling by mitochondria

**Figure 13.**
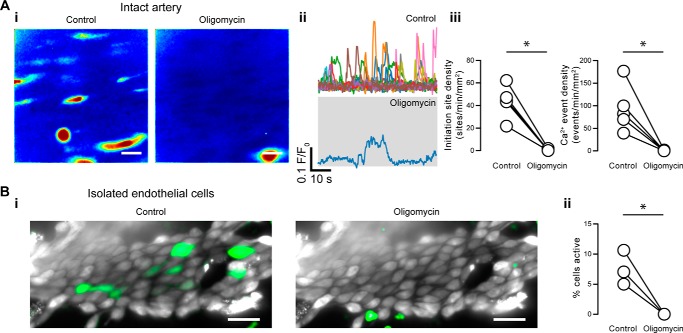
**Mitochondrial ATP facilitates constitutive IP_3_-mediated Ca^2+^ signaling in native endothelium.** *A*, effect of the ATP synthase inhibitor, oligomycin, on basal endothelial Ca^2+^ events in intact arteries. *i*, composite Ca^2+^ images illustrating Ca^2+^ activity (in the same field of endothelial cells) before (*left*) and after (*right*) pharmacological intervention. Images are shown on the same intensity scale; *ii*, Ca^2+^ traces from the events shown in the corresponding panel *i*; *iii*, paired summary data showing the density of Ca^2+^ event initiation sites and Ca^2+^ events. Each data point indicates the mean of three technical replicates (three fields of endothelial cells) from a single experimental unit (one animal). *B*, effect of oligomycin on basal endothelial Ca^2+^ events in isolated sheets of endothelial cells. *i*, raw Ca^2+^ images (*gray*) with Ca^2+^ activity (*green*) overlaid; *ii*, paired summary data showing the percentage of endothelial cells exhibiting basal Ca^2+^ activity. *Scale bars*, 20 μm; *, *p* < 0.05 using paired *t* test.

Taken together, these results suggest that polarized mitochondria are required for IP_3_-mediated, basal endothelial Ca^2+^ dynamics to occur. However, the mean distance between mitochondria and Ca^2+^ event initiation sites measured over short imaging durations, together with the potential for movement of mitochondria, suggest that, rather than tight coupling between the organelle and Ca^2+^ release site facilitating Ca^2+^ buffering, mitochondria may alter endothelial Ca^2+^ signaling via a diffusible factor. Mitochondrial ATP may alter IP_3_R activity (56). However, endothelial cells reportedly rely on glycolysis for ATP production. Regardless, we next examined the effect of inhibiting the mitochondrial ATP synthase using oligomycin alone (6 μm) (*i.e.* without simultaneously uncoupling mitochondria using CCCP or rotenone). Remarkably, we found that inhibition of the ATP synthase nearly eliminated basal Ca^2+^ dynamics (Fig. 13*A*); oligomycin reduced the density of initiation sites from 44 ± 6 to 1 ± 0 sites min^−1^ mm^−2^ and the density of events from 93 ± 23 to 1 ± 0 events min^−1^ mm^−2^ (15 fields from *n* = 5 animals). Oligomycin also inhibited spontaneous Ca^2+^ activity in isolated endothelial cell patches (Fig. 13*B*; *n* = 3).

**Figure 14.**
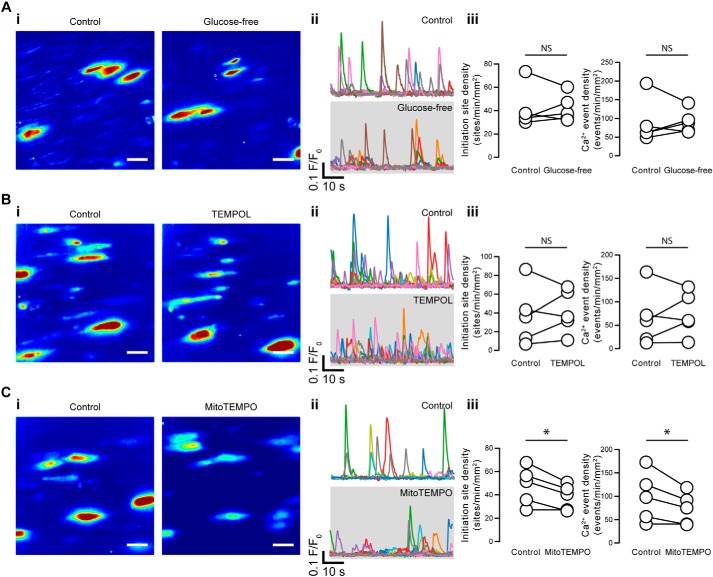
**Basal IP_3_-mediated endothelial Ca^2+^ signaling after removal of by glucose or buffering ROS.** *A–C*, effects of glucose removal (glucose-free; *A*), TEMPOL (*B*), and mitoTEMPO (*C*) on basal endothelial Ca^2+^ events. *i*, composite Ca^2+^ images illustrating Ca^2+^ activity (in the same field of endothelial cells) before (*left*) and after (*right*) pharmacological intervention. Each image pair is shown on the same intensity scale. *Scale bars*, 20 μm. *ii*, Ca^2+^ traces from the events shown in the corresponding panel *i. iii*, paired summary data showing the density of Ca^2+^ event initiation sites and Ca^2+^ events. Each data point indicates the mean of three technical replicates (three fields of endothelial cells) from a single experimental unit (one animal). *, *p* < 0.05; *NS*, no statistically significant difference detected (*i.e. p* > 0.05) using paired *t* test.

Removal of external glucose had no significant effect on either the density of Ca^2+^ event initiation sites (42 ± 8 sites min^−1^ mm^−2^ for control; 42 ± 5 sites min^−1^ mm^−2^ for Ca^2+^-free; *p* = 0.94) or the density of Ca^2+^ events (89 ± 27 events min^−1^ mm^−2^ for control; 91 ± 14 events min^−1^ mm^−2^ for Ca^2+^-free; *p* = 0.90), suggesting that ATP derived from glycolysis is not required to maintain basal endothelial Ca^2+^ dynamics (Fig. 14*A*, 15 fields from *n* = 5 animals). These results suggest that ATP produced by mitochondria, and not glycolysis, facilitates IP_3_-mediated Ca^2+^ signaling in native endothelial cells.

In a final series of experiments, we investigated whether oxidative stress induces basal IP_3_-mediated Ca^2+^ signaling in endothelial cells. To this end, we investigated the effects of the nontargeted, cell-permeable reactive oxygen species (ROS) scavenger, TEMPOL (100 μm), and the mitochondria-targeted ROS scavenger, mitoTEMPO (50 μm). We found that nontargeted ROS scavenging was ineffective in reducing the density of Ca^2+^ event initiation sites (37 ± 14 sites min^−1^ mm^−2^ for control; 42 ± 10 sites min^−1^ mm^−2^ for TEMPOL; *p* = 0.61) or of Ca^2+^ events (66 ± 27 events min^−1^ mm^−2^ for control; 75 ± 21 events min^−1^ mm^−2^ for TEMPOL; *p* = 0.61) in native endothelial cells (Fig. 14*B*). Targeting the mitochondrial site of oxidative stress resulted in a modest decrease (∼20%) in both the Ca^2+^ event initiation site density (∼20%; 48 ± 7 sites min^−1^ mm^−2^ for control; 38 ± 5 sites min^−1^ mm^−2^ for mitoTEMPO) and the Ca^2+^ event density (99 ± 24 events min^−1^ mm^−2^ for control; 73 ± 15 events min^−1^ mm^−2^ for mitoTEMPO) in native endothelial cells (Fig. 14*C*).

## Discussion

**Figure 15.**
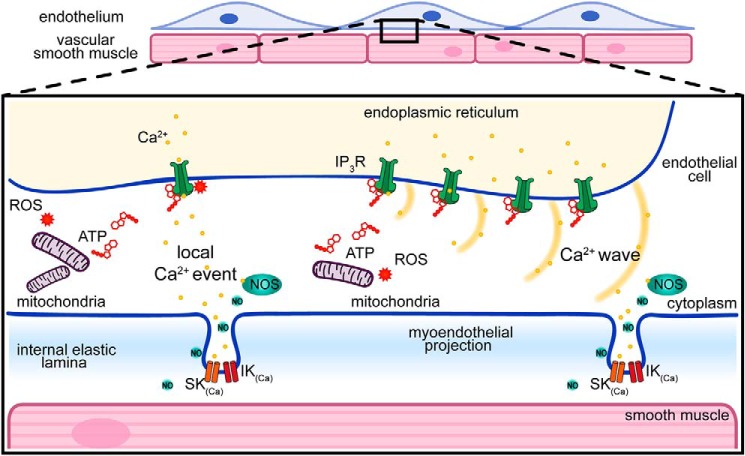
**Model of mitochondrial control of endothelial Ca^2+^ signaling.** Spontaneous local Ca^2+^ events arise from IP_3_ receptor activity. At higher [IP_3_], localized events may trigger waves that propagate across the cell (41, 116). Ca^2+^-dependent effector proteins involved in controlling vascular tone (*e.g.* NOS and IK/SK channels) are enriched at MEPs, and these may be activated by Ca^2+^ events arising from IP_3_Rs located directly at the MEP (by local events) or from IP_3_Rs located some distance away (via propagating Ca^2+^ waves). Mitochondria, by producing ATP and reactive oxygen species, play a key role in maintaining IP_3_-evoked Ca^2+^ dynamics and may act at a distance from the Ca^2+^ release site.

In the present study, we have shown that spontaneous IP_3_-mediated Ca^2+^ signals, in the endothelium of intact small arteries, initiate at sites that are close to MEPs and are controlled by polarized mitochondria. When the mitochondrial membrane potential is depolarized, spontaneous Ca^2+^ signals cease. However, in contrast to many other cell types, mitochondrial control of local signals does not require the organelles to be positioned close to the Ca^2+^ release site. Rather, mitochondria exert long-range control of Ca^2+^ signaling in the vascular endothelium. The most likely candidate for the control is mitochondrial ATP production (Fig. 15).

Among the most important, fundamental functions of mitochondria are the provision of ATP, participation in Ca^2+^ regulation, and generation and elimination of ROS. Each function is driven by the mitochondrial membrane potential (ΔΨm), which is generated by proton pumps (complexes I, III, and IV). Together, the membrane potential and the proton gradient form the transmembrane potential of hydrogen ions used to make ATP. Collapse of the mitochondrial membrane potential inhibits ATP production, prevents mitochondrial Ca^2+^ uptake, and alters the production of ROS. In the present study in native endothelial cells, the uncoupler, CCCP, or the complex I inhibitor, rotenone, each collapsed the mitochondrial membrane potential and inhibited IP_3_-mediated basal Ca^2+^ signaling. Each drug (CCCP or rotenone) was effective in inhibiting Ca^2+^ signals when applied alone or in combination with oligomycin to prevent reversal of the ATP synthase. The ATP synthase blocker oligomycin, applied alone (as expected), did not decrease ΔΨm and yet also inhibited local Ca^2+^ signals. These results suggest that ATP production may maintain local endothelial Ca^2+^ signals in intact arteries. IP_3_-induced Ca^2+^ release may be regulated by a variety of cofactors and processes, which include ATP (71–76). For example, ATP potentiates IP_3_-induced Ca^2+^ release from permeabilized cells and from native endoplasmic reticulum vesicles and enhances activation of IP_3_-gated channels and purified, reconstituted IP_3_ receptor (73–75, 77) by increasing the open time of the channel (78). Inhibiting mitochondrial ATP production appears to abolish the potentiating effect of mitochondria on local IP_3_-evoked Ca^2+^ release in the endothelium.

Mitochondrial Ca^2+^ uptake does not appear to explain mitochondrial maintenance of local IP_3_ Ca^2+^ signals in native endothelial cells. In other cell types, mitochondria may control Ca^2+^ signaling by acting as a Ca^2+^ buffer (79). Close apposition of mitochondria and Ca^2+^ channels is essential to this control (27, 80–83). For example, mitochondria regulate local Ca^2+^ release from IP_3_R clusters (Ca^2+^ puffs) and limit a Ca^2+^-dependent feedback process that controls Ca^2+^ release (40, 84, 85). This mitochondrial control of IP_3_-evoked Ca^2+^ release requires coupling of mitochondria and internal store by tethers that link the organelles close to IP_3_R (36, 86–90). Several candidates for tethers have been identified, such as mitofusin-2 (88), the multifunctional sorting protein PACS-2 (87), σ-1 receptor (90), and the glucose-regulated protein 75 (GRP75) (91). Artificially altering tether lengths or prevention of connections between store and mitochondria has wide-ranging consequence for cell function (36, 87, 88). Maintained apposition of the store and IP_3_ receptors is required because of the low affinity of the uniporter for Ca^2+^ (*K_d_* ∼10–50 μm). The close apposition exposes the organelles to a high local Ca^2+^ concentration as the ion is released from the store, which overcomes the low affinity of the uniporter for Ca^2+^ (63, 92).

In the present study, we observed mitochondria moving throughout the cytoplasm of native endothelial cells. Snapshots of mitochondria revealed that the organelles appeared randomly distributed with respect to Ca^2+^ event initiation sites. Mitochondrial control of local Ca^2+^ signals in native endothelial cells occurred even though the organelles were no denser at Ca^2+^ signal initiation sites than would be expected from a random distribution (Fig. 14). These observations make the possibility that mitochondria were tethered to Ca^2+^ release sites in native endothelial cells unlikely. In line with these observations, mitochondrial dynamics in freshly isolated endothelial cells has been linked to ROS-dependent VEGF production (93).

Mitochondria are also a major source of ROS, and ROS may potentiate endothelial Ca^2+^ release (94). However, ROS does not appear to underlie the present observations. ROS is important in redox signaling from mitochondria to the rest of the cell (95), and physiologically relevant ROS regulates Ca^2+^ signaling by modulating IP_3_R activity (96). Superoxide anions may cause oxidation of the IP_3_ receptor and sensitization of Ca^2+^ release. Various exogenously added oxidants, such as thimerosal (97–99), *t*-butylhydroperoxide (100), and diamide (101, 102), each stimulate IP_3_R-mediated Ca^2+^ release. Mitochondria, by providing a source of ROS, may thus maintain IP_3_-evoked Ca^2+^ release. However, our results show that when ROS is reduced globally by the scavenger, TEMPOL, spontaneous Ca^2+^ release events were unaffected. Targeted scavenging of mitochondrial ROS using the TPP^+^-conjugated form of TEMPOL, mitoTEMPO, resulted in only a modest reduction in endothelial Ca^2+^ activity. Thus, ROS diffusing from mitochondria may also enable the organelles to control IP_3_Rs at significant distances, and the interplay between ATP and ROS may provide a feedback regulation of IP_3_R based on cellular activity.

The structure of mitochondria is believed to be critical in determining precisely how the organelles regulate local and global Ca^2+^ signals in various cell types (59–62). However, relatively little is known of the structure of mitochondria in native endothelial cells. Much of what is known about the structure of mitochondria in endothelial cells has been derived from cultured cells because of the relative ease with which the organelles can be visualized in these cells. In cultured cells, mitochondria exist in a wide range of sizes and shapes, and the organelle may change rapidly from solitary ovoid shapes to extensive branched networks and even to a single continuous mitochondrial structure throughout the cell (27, 103–107). The organelles can also be highly dynamic, continuously reshaping to create a diversity of structures, presumably each with different physiological roles, although the precise functions are not yet fully understood (62, 108). There is much less known about the precise structure of mitochondria cells in fully differentiated endothelial cells. Here, we show that, in small artery endothelial cells, mitochondria exist as spheres, short rods, and relatively small networked sections (as in large artery endothelial cells (45)). The structure is similar to that of native smooth muscle and cardiac cells, perhaps suggesting that mitochondria in fully differentiated cells do not usually form extensive networks (59, 61, 62).

Notwithstanding the absence of a close association with mitochondria, MEPs were associated with spontaneous Ca^2+^ events. Previous studies have also demonstrated co-localization of Ca^2+^ signals and MEPs (42). The definition of co-localization is often ambiguous and varies among studies. In some studies, localization of events within a pixel or voxel is used to define co-localization. However, that in itself may still allow for a large gap between the events under study (see “Discussion” in Ref. 109). In the case of local endothelial Ca^2+^ signals and MEPs, the separation used to define co-localization is often set at 5 μm (110). In the present study, we observed that the mean separation between a Ca^2+^ event initiation site and an IEL hole was ∼2.4 μm. We also performed Monte Carlo simulations to generate randomized data sets for each experiment and analyzed these data. The average minimum distance between an IEL hole and the randomly redistributed Ca^2+^ event initiation sites was 5 μm. Thus, whereas the present results confirm an association between Ca^2+^ release sites and MEPs (42), they also highlight the need for objective criteria in determining thresholds to assess co-localization.

The local Ca^2+^ signals observed near MEPs in the present study were the result of Ca^2+^ release via IP_3_Rs and were unaltered by voltage-dependent Ca^2+^ channel blockers. The Ca^2+^ signals did not have distinguishing features but a continuous range of amplitudes and durations. These findings are similar to the continuum of amplitudes, duration, and spread of local Ca^2+^ signals seen in coronary arteries (111). IP_3_R-mediated Ca^2+^ events that occur near MEPs in mouse mesenteric arteries were reported as being distinctly fast and tightly confined Ca^2+^ changes and were named pulsars to distinguish them from other Ca^2+^ events (42). The reason for the difference in the nature of the signals reported in the present study from those in mouse mesenteric arteries is not completely clear, although differences in species, tissue, or experimental approach may contribute.

Endothelial cells are often considered to contain a small total mitochondrial complement when compared with other energetic cells (10). For example, mitochondria are reported to occupy ∼5% of total cellular volume in endothelial cells, whereas in cardiomyocytes, mitochondria may occupy ∼30% of cell volume (112). This low endothelial mitochondrial content is often cited (*e.g.* see Refs. 5 and 10) as a reason why the role of mitochondria in controlling endothelial physiology has been underestimated. In the present study, ∼9% of the endothelial cell area was occupied by mitochondria, a value comparable (7%) with native smooth muscle cells (59, 60), a cell type in which the role of mitochondria has long been acknowledged.

The results reported in the present study demonstrate that, despite being described as a “glycolytic” cell type, the vascular endothelium requires mitochondrially derived ATP for local spontaneous IP_3_-mediated endothelial Ca^2+^ signaling, which, ultimately, governs vascular tone (42). These findings, together with others (113), demonstrate that the oxidative phosphorylation pathway is required for key endothelial functions and may provide an unexpected route to therapeutic strategies to target endothelial dysfunction.

## Experimental procedures

### Animals

All animal care and experimental procedures were carried out with the approval of the University of Strathclyde Local Ethical Review Panel (Schedule 1 procedure; Animals (Scientific Procedures) Act 1986, United Kingdom), under UK Home Office regulations. All experiments used second- or third-order mesenteric arteries obtained from male Sprague–Dawley rats (10–12 weeks old; 250–350 g), euthanized by overdose of CO_2_.

### Imaging of local endothelial Ca^2+^ signaling

Immediately following euthanasia, the mesenteric bed was removed and placed in PSS composed of 145 mm NaCl, 4.7 mm KCl, 2.0 mm MOPS, 1.2 mm NaH_2_PO_4_, 5.0 mm glucose, 2.0 mm pyruvate, 0.02 mm EDTA, 1.17 mm MgCl_2_, 2.0 mm CaCl_2_, adjusted to pH 7.4 with NaOH. Small mesenteric arteries were then cleaned of connective tissue and fat, removed from the mesenteric bed, cut open using microscissors, and pinned endothelial side-up on a Sylgard block. The endothelium was then preferentially loaded with the fluorescent Ca^2+^ indicator, Cal-520/AM (5 μm with 0.04% Pluronic F127 and 0.26% DMSO in PSS) at 37 °C for 30 min. Following incubation, preparations were gently washed in PSS and mounted in a custom chamber designed for use on an inverted microscope (45, 53, 114). Endothelial Ca^2+^ imaging was then performed at high temporal (20 Hz) and spatial resolution (130-nm projected pixel size at focal plane) using an inverted fluorescence microscope (TE2000U, Nikon, Tokyo, Japan) equipped with a ×100 objective (1.3 NA; Nikon, Tokyo, Japan) and a large-format (1024 × 1024 13-μm pixels) EMCCD camera (iXon 888; Andor, Belfast, UK). Cal-520/AM was excited with 488-nm wide-field epifluorescence illumination provided by a monochromator (Photon Technology International/Horiba UK, Ltd., Stanmore, UK). The resulting image field (∼133 × 133 μm) enabled us to visualize connected networks of ∼50 whole or partial endothelial cells. Following equilibration (30 min), Ca^2+^ activity was recorded at room temperature for periods of 60 s. In experiments investigating the effects of pharmacological intervention, Ca^2+^ recordings were obtained from at least three separate fields of view per animal. Each of these regions was imaged both before and after the intervention. In these experiments, drugs were added directly to the imaging chamber, incubated for 20 min (unless otherwise indicated), and remained present throughout recordings. In experiments using a glucose-free PSS, glucose was substituted with d-mannitol on an equimolar basis. In experiments using a Ca^2+^-free PSS, Ca^2+^ was substituted with Mg^2+^ on an equimolar basis, and 1 mm EGTA was included.

### Imaging Ca^2+^ signaling in sheets of isolated mesenteric artery endothelial cells

Cut open arteries were cut into three or four smaller segments, and the segments were transferred into a PSS solution containing collagenase (2 mg/ml). The segments were incubated in this solution for 20 min at 37 °C and then gently washed three times in PSS. Following the wash steps, endothelial sheets were dispersed by triturating with a fire-polished glass pipette. The suspension was then transferred to an imaging chamber, and isolated endothelial sheets were allowed to attach to the glass coverslip for 1 h. After this period of time, the solution was exchanged for one containing the Ca^2+^ indicator, Cal-520/AM, and loaded at 37 °C for 30 min. The digestion protocol was designed to leave intact sheets of endothelial cells, which could be identified by their morphology (Fig. 5*A*) and by their response to ACh (Fig. 5*B*).

### Analysis of local endothelial Ca^2+^ signaling

Local Ca^2+^ signals recordings were analyzed using a custom semiautomated Python-based analysis adapted from our previous work (45). The procedure for analyzing local Ca^2+^ signals consisted of four parts: 1) preprocessing of Ca^2+^ imaging data; 2) identification of sites of Ca^2+^ activity; 3) extraction of Ca^2+^ signals from active sites; and 4) analysis of Ca^2+^ event parameters. Each step is described below.

#### 

##### Image preprocessing and identification of Ca^2+^ event initiation sites

Ca^2+^-imaging recordings were preprocessed in FIJI as described previously (45). First, to facilitate manual detection of Ca^2+^ events, we created Δ*F/F*_avg_ image stacks by dividing each frame by the mean of all frames. These image stacks were then normalized by dividing each frame in the stack by the S.D. value of all frames, and the resultant stack was further processed by applying a Gaussian blur (2-pixel radius). The preprocessing resulted in an image stack where positive pixel values indicate an increase in fluorescence above the average. Ca^2+^ event initiation sites were then identified manually. To do this, Δ*F/F*_avg_ image stacks were then scrolled through, and each Ca^2+^ event was marked by a circular ROI that was centered over the point of initiation. Events often occurred repeatedly at a single site. In these cases, only a single ROI was positioned over the active site. Each ROI was added to the FIJI ROI manager, and, after ensuring that all Ca^2+^ activity was marked, the center coordinates of the ROIs were saved for subsequent Ca^2+^ signal extraction, as described below.

##### Extraction of Ca^2+^ signals and analysis of Ca^2+^ event parameters

Temporal Ca^2+^ signals were extracted from the raw fluorescence intensity (*F*) image stacks, using 30-pixel (∼4-μm) diameter circular ROIs positioned at the initiation site of each Ca^2+^ event as described above. The signals were extracted and processed using a modification of our previously published algorithm for batch processing of two-dimensional Ca^2+^ data (115). For each initiation site, the intensity values within a 30-pixel (∼3.9 μm) diameter circular ROI were averaged for each frame. Ca^2+^ signals were then smoothed using a 21-point (1.05 s), third-order polynomial Savitzky–Golay filter, corrected for baseline drift using asymmetric least squares fitting,^4^ and differentiated by convolution with the first derivative of Gaussian kernel. Smoothed fluorescence intensity (*F*) traces were expressed as fractional changes from baseline (*F*/*F*_0_) by dividing the fluorescence intensity trace by the average value of a 100-frame (5-s) baseline period (*F*_0_). The baseline period was automatically determined for each trace as the portion of signal exhibiting the lowest S.D. This was achieved by applying a rolling S.D. (100 frames) and a rolling summation (100 frames) to each trace. The minimum of the rolling summation corresponds to the center of the “quietest” portion of the *F*/*F*_0_ trace. Ca^2+^ events were then automatically identified (Fig. 2*A*) using a zero-crossing detector on the derivative *F*/*F*_0_ traces (45). A threshold of 10 times the S.D. of baseline noise was used to distinguish Ca^2+^ events from noise. A positive peak in the derivative function corresponds to the positive edge of a Ca^2+^ event. A negative peak (nadir) in the derivative function corresponds to the negative edge of a Ca^2+^ event. The zero-crossings associated with a peak and a preceding nadir (one before, one between, and one after) in the derivative trace indicate, respectively, the start, peak, and end of an event in the corresponding Ca^2+^ trace. The zero-crossing times were used to extract those parts of the original Ca^2+^ trace that contained Ca^2+^ events. Event parameters (amplitude, FDHM, 10–90% rise time, and 90–10% fall time) were then extracted by fitting each detected Ca^2+^ event with a Gaussian function.

### Assessment of Ca^2+^ event initiation sites and myoendothelial gap junction location

To assess coupling between Ca^2+^ events and myoendothelial gap junctions, images of the internal elastic lamina underlying endothelial cells and Ca^2+^ imaging data were recorded. The IEL was visualized using autofluorescence from 390-nm excitation light and single images generated by averaging 100-frame recordings obtained at 10 Hz. In rat mesenteric arteries, “holes” in the IEL correlate with the presence of myoendothelial gap junctions and may present a low-resistance pathway for diffusible factors (117, 118). To highlight the position of IEL holes, images were smoothed and inverted so that IEL holes appeared as bright regions on a dark background (Fig. 4). IEL hole images were subjected to spatial filtering (2.5-pixel Gaussian kernel) and an automated intensity threshold. Binary Ca^2+^ event initiation site images were created by flooding initiation site ROIs. IEL hole dimensions and IEL hole/Ca^2+^ event initiation site co-localization were then determined using custom-written Python code. The code measured the centroid–centroid distance between every Ca^2+^ event and every IEL hole, and the closest IEL hole to each Ca^2+^ event initiation site was determined.

To determine whether the extent of co-localization of Ca^2+^ event initiation and IEL holes was greater than would be expected if initiation sites and IEL holes were randomly positioned with respect to each other, we used a permutation analysis (119). First, the Ca^2+^ event initiation site data were used to generate a random distribution of initiation sites. Initiation sites within the field of view were randomized, and the location of IEL holes was left unchanged. Co-localization between the randomized Ca^2+^ event initiation sites and the unchanged IEL holes was then calculated as described above. This process was repeated 1000 times for each data set, and a distribution of the minimum (random) initiation site to IEL hole separation was calculated.

### Imaging of endothelial mitochondria

Mitochondria were visualized using the membrane potential–sensitive dye, TMRE (120 nm) (45). TMRE was added to the PSS, and the endothelium was incubated for 10 min before beginning the experiment and was continuously present (120 nm) in all perfusion solutions. Images of TMRE fluorescence (excited at 555 nm) were acquired at 20 Hz using the TE2000U microscope system described above. In experiments assessing mitochondrial morphology, TMRE fluorescence was recorded (1-min periods) from at least three separate fields of view per animal. For each field of view, single images were generated by averaging 100 frames, and mitochondrial morphology was determined using MicroP (120). In experiments that examined the effects of pharmacological intervention on TMRE fluorescence (*e.g.* CCCP, rotenone, or oligomycin), the same single field of endothelial cells was imaged throughout, and TMRE fluorescence was measured after performing a 10-pixel-wide background subtraction using FIJI. In experiments examining mitochondrial motility, mitochondria were imaged for periods of 5 or 30 min at 2 Hz. Mitochondrial movement tracks were generated using TrackMate (121).

### Assessment of mitochondria and Ca^2+^ event initiation sites

To assess the extent of spatial coupling between mitochondria and Ca^2+^ event initiation sites, mitochondrial TMRE fluorescence and Ca^2+^ activity were each recorded in the same field of endothelial cells. Single images of mitochondria were generated by averaging 100 frames (5 s) of the full recording. To facilitate comparison of localization, mitochondria were contrast-enhanced by converting images to a binary form. This was achieved by applying (in sequence) an unsharp mask filter (2-pixel radius), a rolling ball (5-pixel diameter) background subtraction, a mean filter (1-pixel radius), a linear contrast enhancement, an adaptive local contrast enhancement, a median filter (1-pixel radius), and finally an intensity threshold (Otsu's automatic method). The extent of co-localization between mitochondria and the Ca^2+^ event initiation site was then determined as described above for IEL holes.

### Localized photolysis of caged IP_3_

In some experiments, endothelial Ca^2+^ signaling was examined in response to photolysis of caged IP_3_. The endothelium of *en face* arteries was dual-loaded with Cal-520/AM (5 μm), and a membrane-permeant caged IP_3_, caged IP_3_ 4,5-dimethoxy-2-nitrobenzyl (10 μm), 0.02% Pluronic F-127, and 0.35% DMSO in PSS for 30 min at 37 °C. Endothelial Ca^2+^ imaging was then performed at 10 Hz, using an inverted fluorescence microscope (TE300; Nikon, Tokyo, Japan) equipped with a ×40 objective (1.3 NA; Nikon, Tokyo, Japan) and a large-format (1024 × 1024 13-μm pixels) EMCCD camera (iXon 888; Andor, Belfast, UK) with a 325-nm projected pixel size at focal plane. Cal-520/AM was excited with 488-nm wide-field epifluorescence illumination provided by an LED illumination system (PE-300^Ultra^, CoolLED, Andover, UK). The EMCCD was configured to record from the central 512 × 512 pixels, resulting in a field of view of ∼166 × 166 μm (>50 cells visualized). Photolysis of caged IP_3_ was achieved using a xenon flashlamp (Rapp Optoelektronic, Hamburg, Germany) attached directly to the TE300 microscope (51, 116), equipped with a ×40 objective. The photolysis spot size diameter was ∼70 μm. Identical UV flashes in the absence of caged IP_3_ evoked no detectable Ca^2+^ response.

### Data presentation and statistical analysis

Except for probability distributions, the *n* value represents the unit of analysis (number of experimental animals). To create probability distributions, data were pooled from all experimental animals within each treatment group. In general, summary data are presented graphically as individual data points (mean of means within each experimental unit) and in the text as the grand mean with the S.E. indicated. Non-Gaussian data (identified using the D'Agostino–Pearson omnibus test) were log normal. Log normal data were transformed (log_10_), and mean values for each experimental unit were calculated on the logarithmic scale and then back-transformed to their original scale for presentation. In the text, log normal data are presented back-transformed grand means with 95% confidence intervals provided for completeness.

With the exception of experiments assessing the Ca^2+^ response to ionomycin or photolysis of caged IP_3_ or experiments utilizing isolated sheets of endothelial cells, Ca^2+^ imaging data were collected from at least three different fields of endothelial cells from three different arteries per rat. In Ca^2+^ experiments in which ionomycin or caged IP_3_ was used, a single field of endothelial cells was studied per animal. A single field of endothelial cells was also studied (per animal) in experiments examining the effects of CCCP/rotenone on the mitochondrial membrane potential. Data were analyzed using Student's *t* test or repeated measures analysis of variance, with Dunnett's multiple-comparison test, as indicated throughout. All statistical analysis was performed using GraphPad Prism version 6 (GraphPad Software, Inc., La Jolla, CA). A *p* value of <0.05 was considered statistically significant. Data supporting the findings of this study are available from the corresponding authors on request.

## Author contributions

C. W. and J. G. M. developed the concept. C. W., M. D. L., H. R. H., X. Z., and C. B. performed the experiments. C. W. and C. D. S. wrote the analysis software. C. W., C. S., C. B., and J. G. M. analyzed the data. C. W. and J. G. M. drafted the manuscript. C. W., C. D. S., J. M. G., and J. G. M. revised and edited the manuscript. C. W., C. D. S., J. M. G., and J. G. M. sourced funding. All authors approved the final version of the manuscript.

## Supplementary Material

Supporting Information

## Footnotes

4 Eilers P. H., and Boelens, H. F. (2005) Baseline correction with asymmetric least squares smoothing, Leiden University Medical Centre Report.

## Footnotes

3 The abbreviations used are:

EC: endothelial cell
IP_3_: inositol trisphosphate
IP_3_R: inositol trisphosphate receptor
ER: endoplasmic reticulum
MEP: myoendothelial projection
NA: numerical aperture
FDHM: full duration at half-maximum
CPA: cyclopiazonic acid
2-APB: 2-aminoethoxydiphenyl borate
IEL: internal elastic lamina
TMRE: tetramethylrhodamine ethyl ester
CCCP: carbonyl cyanide *p*-chlorophenylhydrazone
PSS: physiological saline solution
ROI: region of interest
RuR: ruthenium red.
