# Supplementary material for: Mitochondrial ATP production provides long-range control of endothelial inositol trisphosphate–evoked calcium signaling
Source: J Biol Chem. 2018 Nov 29;294(3):737–58. doi: 10.1074/jbc.RA118.005913 (PMC6341391; doi:10.1074/jbc.RA118.005913)
Supplement: Supporting Information [file supp_294_3_737__index.html]

Mitochondrial ATP production provides long-range control of endothelial inositol trisphosphate–evoked calcium signaling — Mitochondrial control of endothelial Ca2+ signaling — Mitochondrial ATP production provides long-range control of endothelial inositol trisphosphate–evoked calcium signaling — Mitochondrial control of endothelial Ca2+ signaling — Supporting Information 

# Mitochondrial ATP production provides long-range control of endothelial inositol trisphosphate–evoked calcium signaling

## Supporting Information

- Supporting Information (to be published online) - Supplementary data
- Supporting Information (to be published online) - supplementary movie
- Supporting Information (to be published online) - supplementary movie
- Supporting Information (to be published online) - supplementary movie
