# Supplementary material for: Mitochondrial ATP production provides long-range control of endothelial inositol trisphosphate–evoked calcium signaling
Source: J Biol Chem. 2018 Nov 29;294(3):737–58. doi: 10.1074/jbc.RA118.005913 (PMC6341391; doi:10.1074/jbc.RA118.005913)
Supplement: Supporting Information [file supp_RA118.005913_140858_1_supp_241071_pbbp5y.docx]

**SUPPORTING INFORMATION**

Long-range control of endothelial IP_3_-evoked calcium signaling by mitochondrial ATP production

C**alum Wilson^1^, Matthew D. Lee^1^, Helen R. Heathcote^1^, Xun Zhang^1^, Charlotte Buckley^1^, John M. Girkin^2^, Christopher D. Saunter^2^, and John G. McCarron^1^**

From the Departments of ^1^Strathclyde Institute of Pharmacy and Biomedical Sciences, University of Strathclyde, SIPBS Building, 161 Cathedral Street, Glasgow G4 0RE, UK. ^2^Centre for Advanced Instrumentation, Biophysical Sciences Institute, Department of Physics, Durham University, South Road, Durham, DH1 3LE, UK.

Running title Mitochondrial control of endothelial Ca^2+^ signaling

Correspondence to c.wilson@strath.ac.uk 44 141 548 4976 or [john.mccarron@strath.ac.uk](mailto:john.mccarron@strath.ac.uk) 44 141 548 4119.


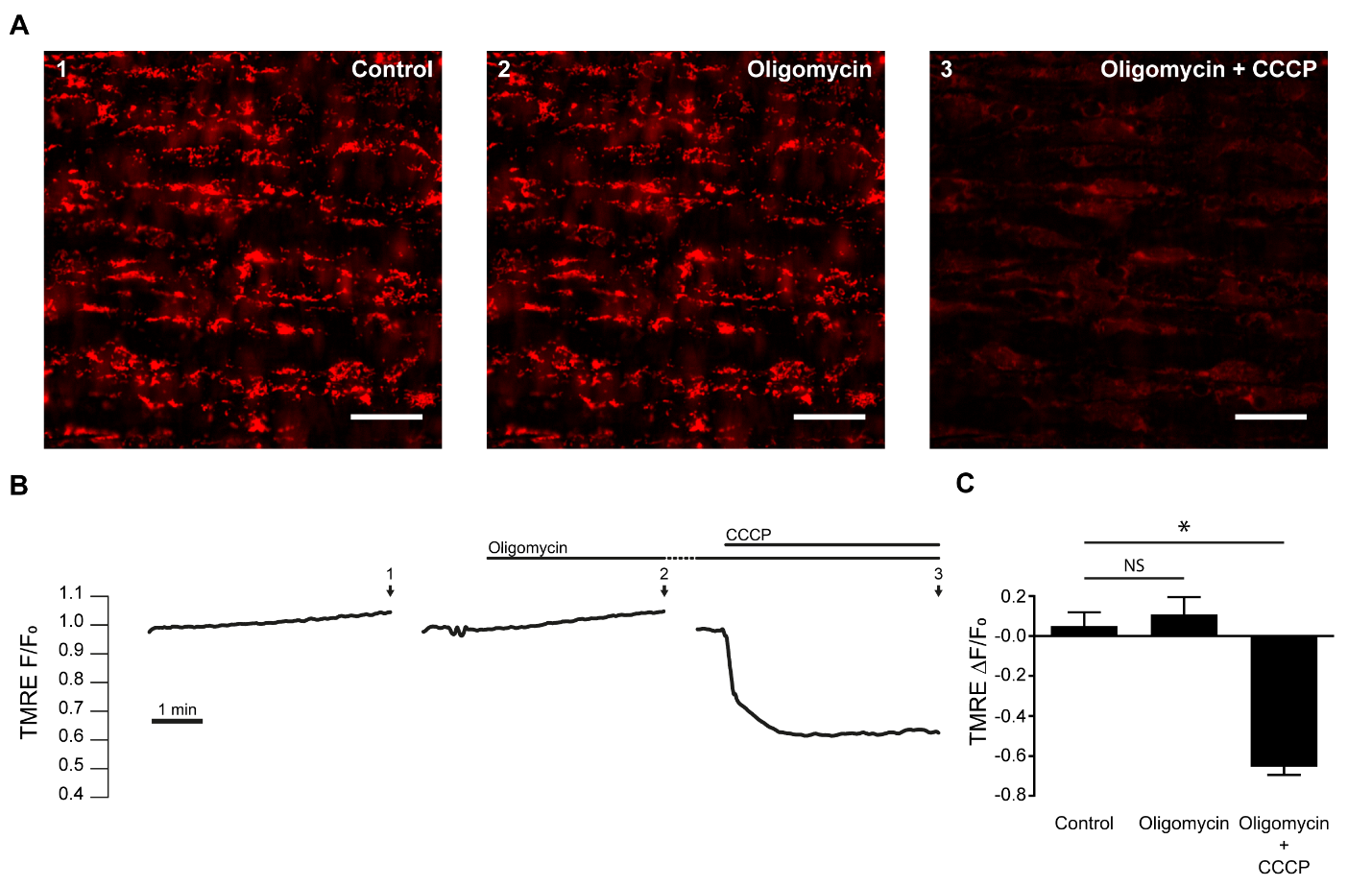


**Figure S1 – CCCP induces mitochondrial depolarization in native endothelial cells.**(A) Representative TMRE images of mesenteric artery endothelium prior to pharmacological intervention (left), in the presence of oligomycin (middle) and in the presence of oligomycin and CCCP (right). Each image is shown on the same intensity scale. Scale bars = 20 µm. B) Traces (F/F_0_) of TMRE fluorescence from the experiment shown in A. C) Summary data illustrating the change in TMRE fluorescence (measured at 5 minutes) in each of the experimental conditions (n = 5). * indicates p<0.05, NS indicates no statistically significant difference detected versus control (i.e. p > 0.05) using repeated measures one-way ANOVA with Dunnetts multiple comparisons test.


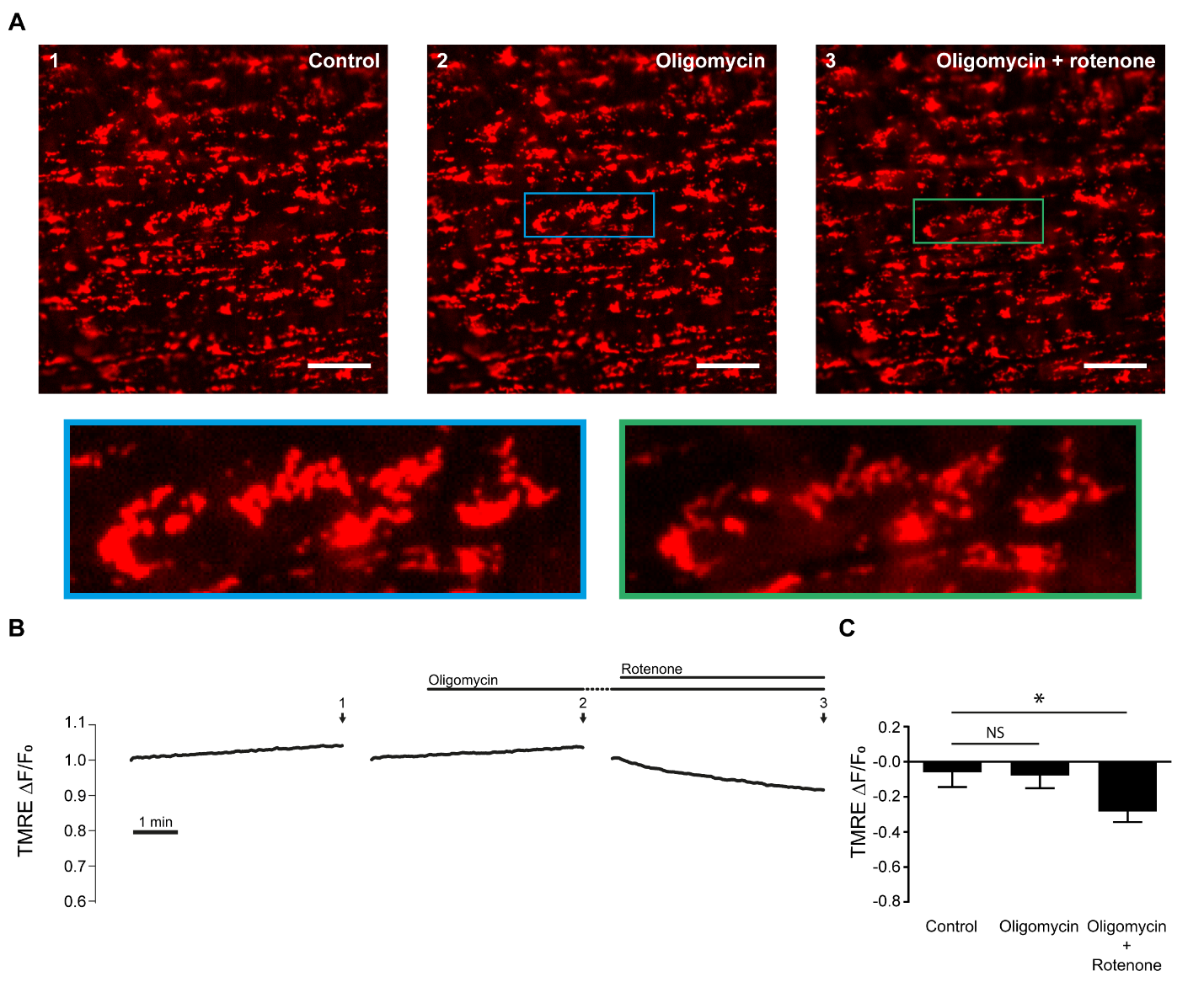


**Figure S2 – Rotenone induces mitochondrial depolarization in native endothelial cells.** (A) Representative TMRE images of mesenteric artery endothelium prior to pharmacological intervention (left), in the presence of oligomycin (middle) and in the presence of oligomycin and rotenone (right). Each image is shown on the same intensity scale. Scale bars = 20 µm. B) Traces (F/F_0_) of TMRE fluorescence from the experiment shown in A. C) Summary data illustrating the change in TMRE fluorescence (measured at 5 minutes) in each of the experimental conditions (n = 3). * indicates p<0.05, NS indicates no statistically significant difference detected versus control (i.e. p > 0.05) using repeated measures one-way ANOVA with Dunnetts multiple comparisons test.


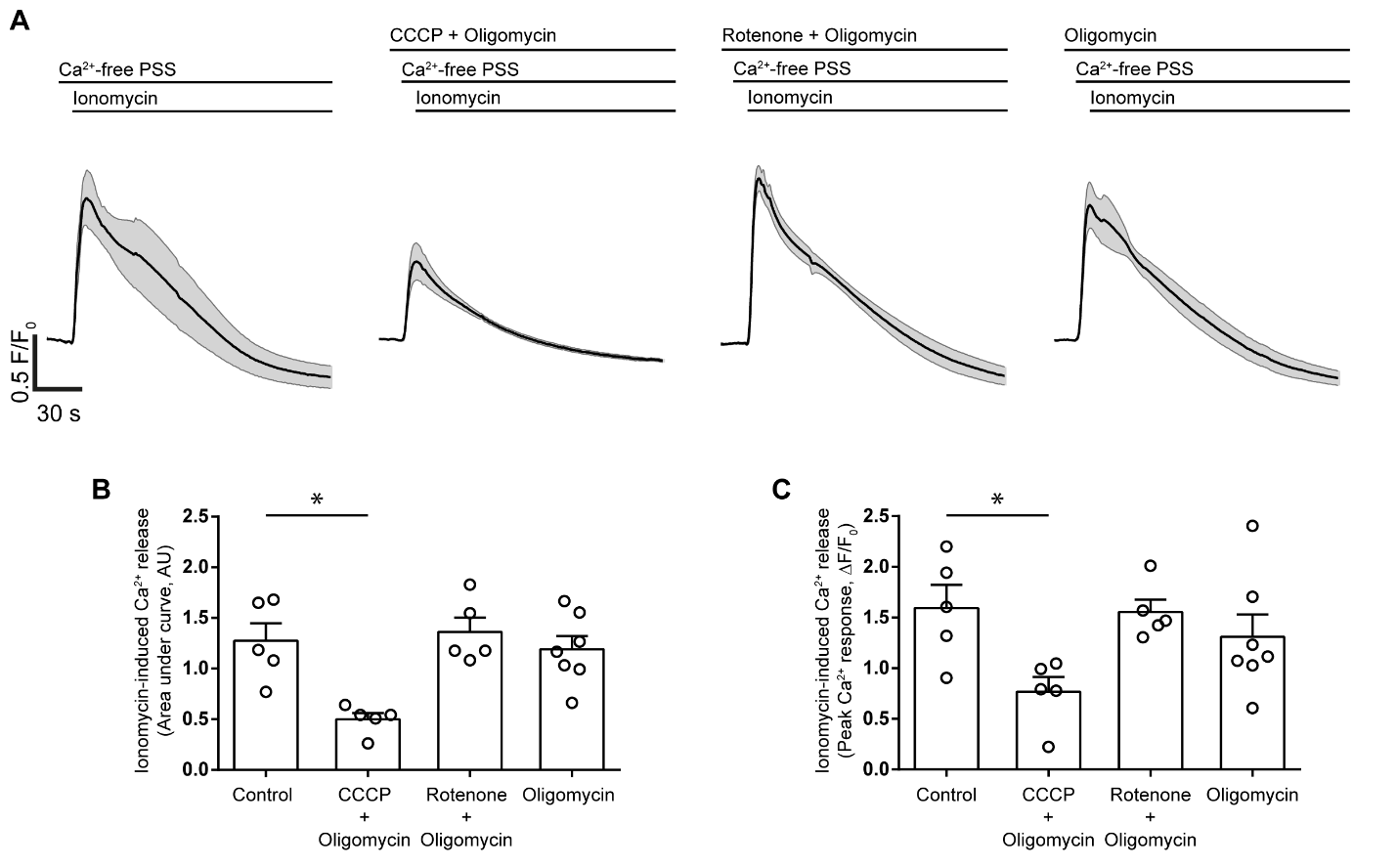


**Figure S3 – Effect of mitochondrial toxins on internal Ca^2+^ store content.** (A) Mean ionomycin-induced (5 µm) Ca^2+^ transients in Ca^2+^ free PSS, in the absence (left-most panel) and presence of various mitochondrial toxins (other panels). Each trace (black line) represents the mean of n > 5 independent trials, the shaded (grey) error bands represent the standard error of the mean. Ca^2+^ responses were averaged over the entire field of view. (B-C) Summary data illustrating the area under the curve (B) and the peak (C) of the ionomycin-induced Ca^2+^ response. * indicates p<0.05, versus control (i.e. p > 0.05) using repeated measures one-way ANOVA with Dunnetts multiple comparisons test
